# Long‐term exposure to excessive norepinephrine in the brain induces tau aggregation, neuronal death, and cognitive deficits in early tau transgenic mice

**DOI:** 10.1111/acel.14420

**Published:** 2024-11-26

**Authors:** June‐Hyun Jeong, Dong Kyu Kim, Sunwoo Chung, Jong Won Han, Jihui Han, Inhee Mook‐Jung

**Affiliations:** ^1^ Department of Biochemistry and Biomedical Sciences, College of Medicine Seoul National University Seoul Korea; ^2^ Convergence Dementia Research Center, College of Medicine Seoul National University Seoul Korea; ^3^ Neuroscience Research Institute, Medical Research Center, College of Medicine Seoul National University Seoul Korea

**Keywords:** Alzheimer's disease, neurodegeneration, norepinephrine, organoid, tau

## Abstract

Alzheimer's disease (AD) is marked by the presence of intraneuronal neurofibrillary tangles (NFTs), which are primarily composed of hyperphosphorylated tau protein. The locus coeruleus (LC), the brain's main source of norepinephrine (NE), is one of the earliest regions to develop NFTs and experience neurodegeneration in AD. While LC‐derived NE plays beneficial roles in cognition, emotion, locomotion, and the sleep–wake cycle, its impact on tau pathology is unclear. To explore this relationship, we administered intraperitoneal injections of either N‐(2‐chloroethyl)‐N‐ethyl‐2‐bromobenzylamine (DSP4), a selective neurotoxin for noradrenergic neurons, or reboxetine (RBX), a norepinephrine reuptake inhibitor, to decrease or increase NE levels, respectively, in early tau transgenic mice expressing mutant human P301L tau (ADLP^Tau^) for two months. Only the RBX‐treated mice exhibited cognitive deficits, as evidenced by their performance in the Y‐maze, novel object recognition, and contextual fear conditioning tests. Immunohistochemical analysis revealed increased hyperphosphorylated tau aggregates in the LC and hippocampus of the RBX‐treated mice. Furthermore, neuronal apoptosis was observed in the hippocampal CA1 region of these mice. Western blotting showed that RBX injections led to the overactivation of tau kinases PKA and GSK3β, resulting in hyperphosphorylated tau, neuronal loss, and cognitive impairments. Consistent with these findings, human brain organoids exposed to higher NE concentrations also displayed elevated hyperphosphorylated tau and increased activity of the same tau kinases. These findings suggest that excessive NE exposure accelerates tau pathology by overactivating the tau kinases. Thus, modulating NE levels in the brain via the LC‐NE system could be a potential therapeutic strategy for tau‐related AD.

## Graphical abstract

Prolonged treatment with reboxetine (RBX) increased norepinephrine (NE) levels in the brains of ADLP^Tau^ mice, significantly accelerating tau aggregation. Severe tau aggregation, resulting from sustained elevated NE levels in the brain, induced neurodegeneration and cognitive deficits. Elevated NE levels in the brain overactivated two well‐known tau kinases, PKA and GSK3β, leading to an imbalance in tau phosphorylation that promotes hyperphosphorylated tau and subsequent tau aggregation.

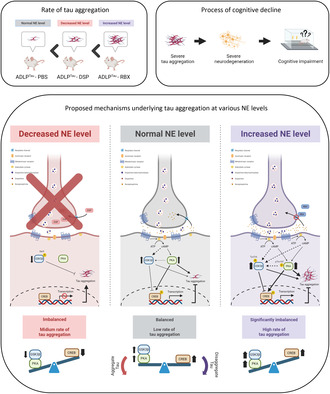

**Abbreviations**
AD: Alzheimer's Disease
ADLP: AD‐Like Pathology
APP: Amyloid‐beta Precursor Protein
CREB: Cyclic‐AMP Response Element‐Binding Protein
DBH: Dopamine Beta‐Hydroxylase
DSP4: N‐(2‐chloroethyl)‐N‐ethyl‐2‐bromobenzylamine
EB: Embryoid Body
GSK3β: Glycogen Synthase Kinase 3 beta
hiPSCs: Human‐induced Pluripotent Stem Cells
IHC: Immunohistochemistry
IP: Intraperitoneal
LC: Locus Coeruleus
NE: Norepinephrine
NFTs: Neurofibrillary Tangles
PKA: Protein Kinase A
RBX: Reboxetine

## 1 INTRODUCTION

Alzheimer's disease (AD) is the most common type of dementia, characterized by extracellular amyloid beta plaques and intracellular neurofibrillary tangles (NFTs) in the brain (Querfurth & LaFerla, 2010). AD is also marked by neuronal loss, synaptic dysfunction, gliosis, and memory impairments (De Sousa, 2022; Griffiths & Grant, 2022). Despite intense research into late‐stage AD, which already possesses most of the pathologic hallmarks, remarkable therapies have not emerged. As a result, many researchers are shifting their attention to early‐stage AD to prevent or delay the initial state of the disease. We have also explored various methods to identify biomarkers for diagnosing early AD (Han et al., 2019; Lee et al., 2022; Park et al., 2019). In this study, our focus is on the locus coeruleus (LC) and tau pathology in the early stages of AD because hyperphosphorylated tau appears in the LC before it does in any other area of the brain (Braak et al., 2011).

The LC, composed of mostly medium‐sized neurons in the pons of the brainstem, is the brain's sole source of norepinephrine (NE), transporting it throughout the brain in response to stress or arousal (Benarroch, 2009). NE in the brain not only controls numerous cognitive activities such as attention, motivation, mood, and memory (Benarroch, 2009) but also regulates blood–brain barrier permeability, glial cell activity, and neuronal metabolism (Mravec et al., 2014). Additionally, NE provides neuroprotection during neuroinflammatory processes (Feinstein et al., 2002). Thus, maintaining normal NE levels in the brain is crucial for these beneficial roles.

Observations from postmortem studies, patient examinations, and animal models of AD indicate that LC integrity is negatively correlated with AD. This suggests that reduced NE levels, due to the loss of noradrenergic innervation to the brain, accelerate the development or progression of AD (Jacobs et al., 2021; Chalermpalanupap et al., 2017). Consequently, several NE‐elevating drugs, including norepinephrine reuptake inhibitors, monoamine oxidase inhibitors, α2 adrenergic antagonists, and L‐DOPS, have been developed to target individuals with AD who experience decreasing NE levels in the brain (Gutiérrez et al., 2022).

Although the therapeutic effects of NE have been researched for decades, some recent studies have shown that abnormally high NE levels in the brain may be harmful in AD. Stress increases NE levels in the brain by activating the LC, and excessive NE release causes cognitive impairments (Finlay et al., 1995). Because of the compensatory mechanism, some AD patients have greater NE levels than normal, which are associated with cognitive problems (Gannon & Wang, 2019). In addition, chronic stress could exacerbate amyloid beta accumulation, tau hyperphosphorylation, neurodegeneration, and cognitive deficits in AD model mice (Carroll et al., 2011). Finally, recent research suggests that NE metabolites may lead to the death of noradrenergic neurons in the LC, where NE is abundant. According to the study, NE can be converted to the toxic metabolite 3,4‐dihydroxyphenyl glycolaldehyde (DOPEGAL), which activates the asparagine endopeptidase (AEP) that facilitates tau tangles followed by neurodegeneration (Kang et al., 2019). In a subsequent investigation, DOPEGAL can bind a tau protein directly and accelerate its aggregation and propagation (Kang et al., 2022).

To effectively treat AD with NE‐related medications, a deeper understanding of the intricate roles of NE is required. Based on the previous studies, we believe that both abnormally high and low NE levels in the brain can induce tau pathology. To examine this hypothesis, we modified NE levels in early tau transgenic mice for a long time by injecting either N‐(2‐chloroethyl)‐N‐ethyl‐2‐bromobenzylamine (DSP4), a selective neurotoxin for noradrenergic neurons, to deplete NE levels, or reboxetine (RBX), a norepinephrine reuptake inhibitor, to increase NE levels. We then assessed whether long‐term alterations in NE levels in both directions could cause behavioral abnormalities using open field tests, Y‐maze, novel object recognition, and contextual fear conditioning tests. Following that, we performed immunohistochemistry and western blotting to investigate other pathological features of tauopathy, such as tau aggregation and neurodegeneration. Furthermore, we explored which tau kinases and/or phosphatases were modulated by the drugs, affecting hyperphosphorylated tau and tau aggregation.

Ultimately, we verified whether human‐derived systems were capable of generating outcomes that were equivalent to the data obtained from mice. We used human brain organoids and exposed them to higher quantities of NE in order to replicate the elevated NE levels reported in the RBX mice. Next, we investigated to see if these organoids displayed comparable pathological characteristics, such as tau pathology and the activation of tau kinases, using western blotting analysis.

## 2 METHODS

### 2.1 Animals

Tau transgenic mice (ADLP^Tau^) and wild‐type mice (ADLP^WT^) were come from the newly created model, called ADLP (AD‐like pathology) animal model (Kim et al., 2018). Young adult female mice of them were used for all experiments. The mice were kept under a 12 h light/dark cycle and given food and water ad libitum. All behavioral tests were performed during the light phase of the cycle. All procedures were overseen under the regulations from the Institutional Animal Care and Use Committee (IACUC).

### 2.2 Drug administration

N‐(2‐chloroethyl)‐N‐ethyl‐2‐bromobenzylamine short for DSP4 (C8417, Sigma), reboxetine mesylate short for RBX (ab120157, Abcam), and phosphate buffered saline short for PBS (P38135‐10PAK, Sigma) were utilized for intraperitoneal (IP) injection. All tau transgenic mice except the wild‐type (WT) mice group were injected once a day except Sundays for two months. The injections were given alternately to the left or right abdominal cavity to prevent any inflammation which could occur due to daily injection to the same spot. Each injection was applied with 100 μL of DSP4 (50 mg/kg), RBX (10 mg/kg), or PBS. The mice treated with PBS or RBX were daily injected. However, the mice with DSP4 were injected with mostly PBS except the days on which DSP4 was treated because it was enough to apply DSP4 once a month to reduce norepinephrine level in the brain.

### 2.3 Behavioral analysis

#### 2.3.1 Open field test

Mice were placed in the opaque Plexiglas square chamber (40 cm × 40 cm) to freely explore for 30 min while the video for the mice was recorded. The video was analyzed with EthoVision XT (Noldus Information Technology, Leesburg, VA, USA). With the software, total distance moved and velocity were automatically measured.

#### 2.3.2 Y‐maze spontaneous alternation test

Mice were introduced to the center of a Y‐shaped maze with three white and opaque plastic arms at a 120° angle from each other. The mice were allowed to explore the arms for 8 min while the video was recorded. The total number of entries and the number of alternations were counted manually.

#### 2.3.3 Novel object recognition test

Mice were habituated to the experimenter's hands for 5 min and then moved to the opaque Plexiglas square chamber (40 cm × 40 cm) for 10 min on two consecutive days. On the training day, the mice were exposed to the same chamber for 10 min in which two identical objects were positioned in the back corners of the arena. After 24 h, the mice were re‐exposed to the environment for 5 min in which one of the two objects was replaced by a new or novel object. The training and test sessions were recorded by a video camera (Canon). Nose touches to the objects or orientation toward the objects within 1 cm were scored manually because automated measurements falsely counted inattentive moments of walking around the objects. The behavioral analysis was performed blind.

#### 2.3.4 Contextual fear conditioning test

Mice were habituated to the experimenter's hands for 5 min on three consecutive days. On the conditioning day, mice were conditioned with a 0.5 mA shock of 2 s duration after 3 min exploration in the Coulbourn chamber (Coulbourn Instruments, Holliston, MA, USA). After the shock, the mice were held for a further 30 s and put back into their home cages. After 24 h, the mice were placed in the same chamber for 3 min. The training and test sessions were recorded by a video camera (Canon). Freezing levels were recorded manually. The behavioral analysis was performed blind.

### 2.4 Serum collection

Mice were anesthetized by inhalation of isoflurane during blood collection. Blood was collected from retro‐orbital bleeding by inserting a capillary tube into the medial canthus of the eye. The blood sample was allowed to clot by leaving it undisturbed at room temperature. After 30 min, the serum was obtained by centrifuging at 2000 ***g*** at 4°C for 15 min. The serum was stored at −80°C until use.

### 2.5 Brain preparation

Mice were anesthetized with a mixture of Zoletil 50 (Virbac, Carros, France) and Rompun (Bayer, Leverkusen, Germany) at a ratio of 3:1. The mice were perfused with cold PBS. Then, each brain was taken out from a skull, and then a hippocampus from one hemisphere was isolated and stored at −80°C until further western blot or immunoassay analysis. The other hemisphere was fixed in 4% paraformaldehyde (PFA) for 24 h at 4°C, transferred to 30% sucrose (w/v) in PBS for 48 h, and stored at −80°C until further immunohistochemistry analysis.

### 2.6 Enzyme immunoassay for norepinephrine

Norepinephrine levels of serum and hippocampus were analyzed by using a norepinephrine high‐sensitive ELISA kit according to the manufacturer's protocol (EA633/96, DLD Diagnostika GmbH). Serum and hippocampal samples were prepared for the ELISA by following the sections of the protocol titled plasma and tissue samples, respectively.

### 2.7 Cell culture and transfection for GSK3β activity analysis

HEK293FT cells were cultured in Dulbecco's Modified Eagle's Medium (DMEM; HyClone) supplemented with 10% fetal bovine serum, 100 U/mL penicillin, and 0.1 mg/mL streptomycin. Once the cells reached sufficient confluency in 100 mm dishes, they were transferred and plated at a density of 1 × 10^5^ cells/ml in 12‐well plates. The following day, the cells were transfected with plasmids encoding GSK3β WT (Addgene, #14753), GSK3β S9A (Addgene, #14754), or GSK3β K85A (Addgene, #14755) using Lipofectamine LTX (Thermo Fisher Scientific), following the manufacturer's protocol. All transfections were performed in triplicate. On day 2, to investigate the effect of norepinephrine (NE) on GSK3β activity, the transfected cells were treated with 100 μM NE in serum‐free DMEM for 24 h.

### 2.8 Human brain organoids generation

To generate human brain organoids from human‐induced pluripotent stem cells (hiPSCs), the BIONi010‐C‐2 cell line purchased from Bioneer was utilized following established protocols (Park et al., 2021). Initially, the hiPSCs were detached from culture plates using ReLeSR (STEMCELL Technologies) and collected by centrifugation. The resultant pellet was then dissociated in EB formation medium (STEMCELL Technologies) containing the ROCK inhibitor, Y‐27632 (STEMCELL Technologies), and plated in AggreWell800 plates (STEMCELL Technologies) to facilitate embryoid body (EB) formation. On the following day (day 1), the medium was changed to EB formation medium without Y‐27632. From day 2 to day 5, the medium was replaced daily with DMEM/F‐12 supplemented with GlutaMAX (Gibco), 20% KnockOut Serum Replacement (Gibco), 1% MEM Non‐Essential Amino Acids Solution (Gibco), 0.1 mM 2‐mercaptoethanol (Gibco), 100 U/mL penicillin, 100 μg/mL streptomycin (Merck), and SMAD inhibitors, dorsomorphin (Merck) at 10 μM and SB‐431542 (TOCRIS) at 10 μM. On day 6, the embryoid bodies were collected and individually transferred to wells of a 96‐well ultra‐low‐attachment microplate (Corning) to undergo further development into brain organoids. These organoids were cultured in neural differentiation medium (Neurobasal‐A Medium; Gibco) supplemented with B‐27 supplement (minus vitamin A; Gibco), P/S, GlutaMAX, and 0.5% (v/v) Matrigel Basement Membrane Matrix (Corning), along with epidermal growth factor (EGF; Merck) at 20 ng/mL and basic fibroblast growth factor (bFGF; R&D Systems) at 20 ng/mL. Fresh neural differentiation medium was replaced daily until day 15, then every other day between days 16 and 24. From day 25 to 42, the medium was substituted with neurobasal medium supplemented with brain‐derived neurotrophic factor (BDNF; Peprotech) at 20 ng/mL and neurotrophin‐3 (NT‐3; Peprotech) at 20 ng/mL every two days to promote neuronal maturation. After day 43, the brain organoids were kept in neurobasal medium with no growth factors, and the medium was renewed every four days until the organoids were completely developed. Over day 100, the organoids containing suitable expansion of astrocytes as well as neurons were treated with various concentrations of norepinephrine (NE) in Opti‐MEM (Gibco) for 24 h.

### 2.9 Western blot analysis

Frozen hippocampus tissues, live transfected cells, or live human brain organoids were homogenized in RIPA buffer supplemented with phenylmethylsulfonyl fluoride (Sigma), protease inhibitor cocktail (Sigma), phosphatase inhibitor cocktail I, and II (AG Scientific). The RIPA‐soluble samples were incubated with 4x NuPAGE sample buffer (Thermo Fisher Scientific) and 10x NuPAGE sample reducing agent (Thermo Fisher Scientific) diluted in DW at 70°C for 10 min for denaturing. The samples were loaded and separated on 4%–12% Bis‐Tris NuPAGE gradient gels (Thermo Fisher Scientific) in MES buffer (Thermo Fisher Scientific). The proteins from the gels were transferred to PVDF membranes (Millipore, Massachusetts, USA) and blocked with 5% skim milk in TBST (TBS with 0.05% Tween‐20) for 1 h followed by overnight incubation of primary antibodies at 4°C. After incubation, the membranes were incubated with horseradish peroxidase (HRP) secondary antibodies (goat anti‐mouse HRP conjugated (1:5000, #31430, Thermo Fisher Scientific) and goat anti‐rabbit HRP conjugated (1:5000, #31460, Thermo Fisher Scientific)) for 1 h at room temperature. The proteins were detected with ECL Western Blotting Detection Reagents (AbFrontier, Seoul, Korea) and imaged by using Amersham Imager 600 (GE Healthcare, Uppsala, Sweden). The western blots were analyzed with Multi Gauge V3.0 (FUJIFILM, Tokyo, Japan). The primary antibodies used for western blotting were Tau13 (1:2000, ab19030, Abcam), AT8 (1:1000, MN1020, Thermo Fisher Scientific), AT180 (1:1000, MN1040, Thermo Fisher Scientific), pS262 (1:1000, 44‐750G, Thermo Fisher Scientific), pS409 (1:1000, 44‐760G, Thermo Fisher Scientific), Dopamine beta‐hydroxylase (1:500, ab209487, Abcam), 6E10 (1:1000, #803001, Biolegend), PKA (1:1000, #4782, Cell Signaling Technology), pPKA‐T197 (1:1000, #4781, Cell Signaling Technology), GSK3β (1:1000, #12456, Cell Signaling Technology), pGSK3β‐Y216 (1:1000, BD612312, BD sciences), pGSK3β‐S9 (1:1000, #9322, Cell Signaling Technology), p38 (1:1000, #9212, Cell Signaling Technology), p‐p38‐T180/Y182 (1:1000, #4511, Cell Signaling Technology), ERK (1:1000, #4695, Cell Signaling Technology), pERK‐T202/Y204 (1:1000, #9101, Cell Signaling Technology), Akt (1:1000, #9272, Cell Signaling Technology), pAkt‐S473 (1:1000, #9271, Cell Signaling Technology), CaMKII (1:1000, ab52476, Abcam), pCaMKII‐T286 (1:1000, #12716, Cell Signaling Technology), p35/25 (1:1000, #2680, Cell Signaling Technology), PP1 (1:1000, #2581, Cell Signaling Technology), pPP1 (1:1000, #2582, Cell Signaling Technology), PP2A (0.1 μg/mL, AF1653, R&D Systems), pPP2A (1 μg/mL, AF3989, R&D Systems), and β‐Actin (1:2000, #3700, Cell Signaling Technology).

### 2.10 Immunohistochemistry analysis (mouse brain tissues)

Frozen hemisphere was covered with FSC 22 Compound (3,801,480, Leica) and coronally sectioned to 30 μm thickness using CM1850 cryostat (Leica, Wetzlar, Germany) and stored in tissue storage buffer at 4°C until use. At room temperature (RT), the tissue slices containing hippocampus were sequentially immersed in 70% formic acid in PBS for 20 min and blocking solution (5% horse serum, 0.5% BSA, 0.3% triton X‐100 in PBS) for 1 h. Then, the blocked tissues were incubated with primary antibody diluted in the blocking solution for overnight. On the next day, the samples were incubated with secondary Alexa® fluorophore‐conjugated antibodies (1:500) diluted in the solution of 5% horse serum in PBS for 1 h in the dark at RT. When immunofluorescence experiments required Congo Red staining, Congo Red (C6277, Sigma) dissolved in DW was added to the secondary antibody solution during the incubation step. Lastly, those were washed 3 times with PBS and co‐stained with DAPI (1:5000) for 5 min right before mounted on a slide. If necessary, signal amplification was performed by additional incubation with biotinylated antibody for 1 h followed by Alexa® fluorophore‐conjugated Streptavidin (1:500). The mounted cells were imaged by LSM700 (Carl Zeiss, Oberkochen, Germany) and analyzed with ImageJ software. The primary antibodies used in the experiments were Tau13 (1:200, #835201, BioLegend), AT8 (1:200, MN1020, Thermo Fisher Scientific), AT180 (1:200, MN1040, Thermo Fisher Scientific), NeuN (1:500, #24307, Cell Signaling Technology), Cleaved Caspase‐3 (1:100, #9661, Cell Signaling Technology), Dopamine beta‐hydroxylase (1:400, ab209487, Abcam), Iba1 (1:500, 019–19,741, FUJIFILM Wako Pure Chemical Corporation), and GFAP (1:1000, 13–0300, Thermo Fisher Scientific).

### 2.11 Immunohistochemistry analysis (human brain organoids)

The procedure began with washing live human brain organoids using PBS, followed by immersion in 4% paraformaldehyde (PFA) for 24 h at 4°C to fixate the tissue. Subsequently, the fixed organoids were transferred to 30% sucrose (w/v) in PBS for cryoprotection over 48 h at 4°C. The organoids were moved to a Biopsy Cryomold (4565, SAKURA), stained with Trypan Blue solution (93,595, Sigma), molded with FSC 22 Compound (3,801,480, Leica), and stored at −80°C until immunohistochemistry analysis. The visualized organoids were sectioned to 30 μm thickness using a CM1850 cryostat (Leica, Wetzlar, Germany) and mounted on slices immediately. Before rinsing the organoids, the organoids on slices were boundary with ImmeEdge Pen (H‐4000, Vector Laboratories) in order to prevent solutions from spreading out of the slices. The organoids were washed three times with PBS in the boundary using pipetting. Then, they were permeabilized using 0.3% PBST (0.3% Triton X‐100 in PBS) for 30 min at room temperature (RT). After 30 min, they were immersed in blocking solution (5% horse serum in PBS) for 1 h at RT. They were applied and incubated with primary antibodies diluted in the blocking solution at 4°C overnight. The next day, primary antibodies were removed and washed three times with PBS. Secondary antibodies were diluted at a ratio of 1:1000 in 5% BSA in 0.3% PBST and treated for 1 h in the dark at RT. Slices were washed three times with PBS and co‐stained with DAPI (1:5000) for 5 min right before covered with cover glass in mounting medium (#MO1, biomeda). The mounted tissues were imaged by LSM700 (Carl Zeiss, Oberkochen, Germany). The primary antibodies used for this experiment were AT8 (1:400, MN1020, Thermo Fisher Scientific), D54D2 (1:300, #8243, Cell Signaling Technology), MAP2 (1:500, ab5392, Abcam), and GFAP (1:300, 13–0300, Thermo Fisher Scientific).

### 2.12 Statistical analysis

Experimental data were analyzed statistically using GraphPad Prism 8 software. All data were shown as mean ± SEM. Statistical significance was determined by unpaired *t* tests or one‐way ANOVA. The statistical significance was denoted by *p < 0.05, **p < 0.01, ***p < 0.001, or ****p < 0.0001.

## 3 RESULTS

### 3.1 Both DSP4 and RBX can influence the amount of NE in the hippocampus of ADLP^Tau^ mice

Before investigating whether tau pathology in the ADLP^Tau^ mice could be accelerated or delayed by altering norepinephrine (NE) levels, we examined the effectiveness of two drugs, N‐(2‐chloroethyl)‐N‐ethyl‐2‐bromobenzylamine (DSP4) and reboxetine (RBX), in the tau transgenic mice. Since DSP4 and RBX had own mechanism of action (Ross & Stenfors, 2014; Wong et al., 2000), we administered DSP4 through intraperitoneal (IP) injection in advance. While injecting DSP4 to a test group, we also injected the other comparison groups with phosphate buffered saline (PBS) to mitigate injection‐related side effects. Subsequently, we injected RBX into another test group the day before and on the day of serum or hippocampus collection. While having RBX injection to the test group, we injected PBS to the other groups for the same reason. We allowed the mice to habituate for an hour after the injection and obtained serum or hippocampus (Figure S1A). Using a highly sensitive NE ELISA kit, we confirmed that DSP4 decreased NE levels, but RBX increased them in the hippocampus (Figure S1B). Importantly, both drugs did not affect NE levels in the serum collected from the blood (Figure S1C). Given that these drugs target noradrenergic neurons in the central nervous system (Ross & Stenfors, 2014; Wong et al., 2000), we demonstrated that they influenced NE levels in the brain, specifically in the hippocampus, and not in the serum.

### 3.2 Only the ADLP^Tau^ mice treated with RBX show cognitive impairment

**FIGURE 1:**
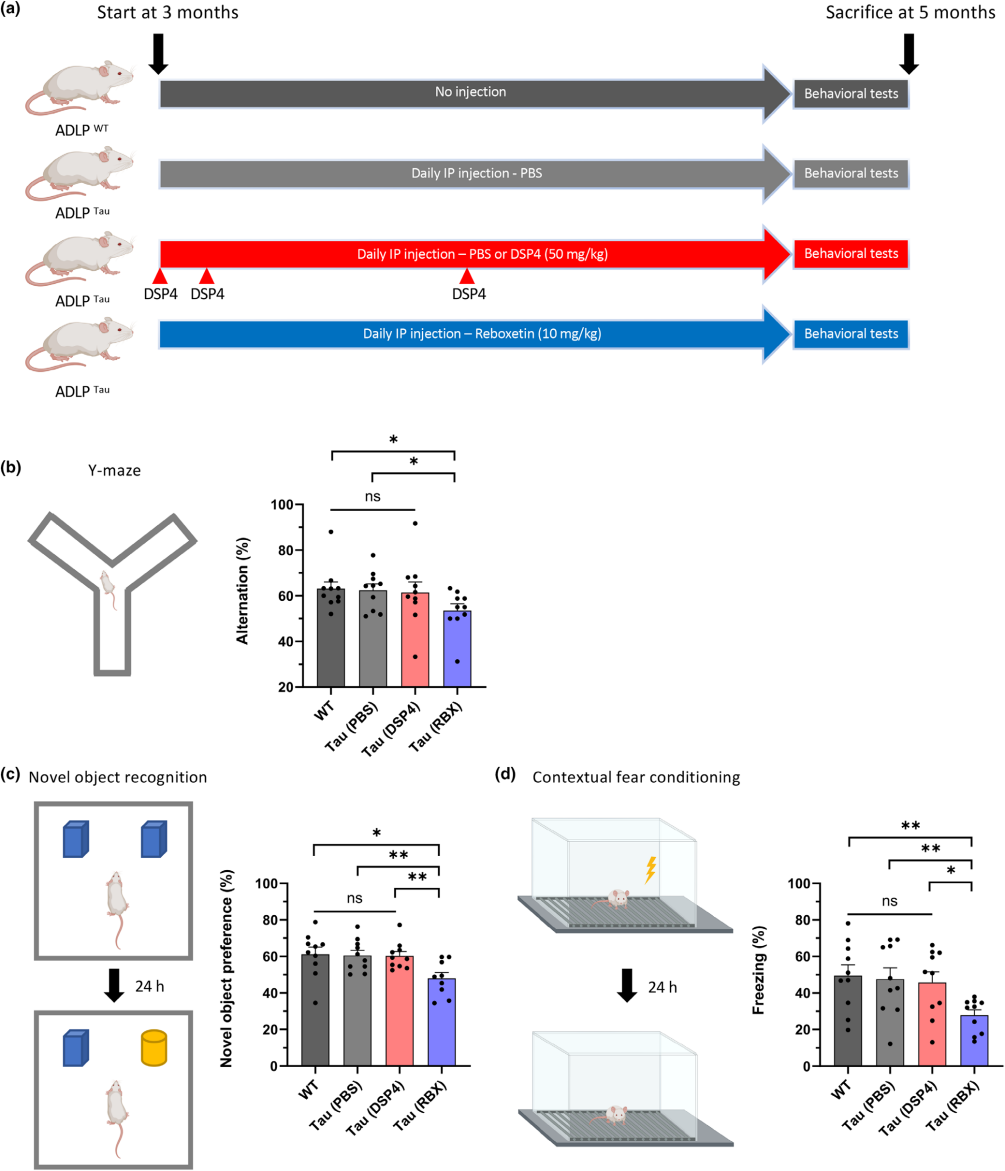
The only ADLP^Tau^ mice given RBX treatment experience cognitive impairment. (a) Schematic representation of the experimental design of 3‐month‐old mice and drug treatment for 2 months. (b) Percentage of spontaneous alternations in the Y‐maze task. (c) Percentage of novel object preference in the novel object recognition test. (d) Percentage of freezing in the contextual fear conditioning test. One dot in the bar graphs represents each mouse (b–d). All data represent mean ± SEM. Significance was determined by unpaired *t* test and denoted by *p < 0.05, **p < 0.01, ***p < 0.001, or ****p < 0.0001.

We initiated an animal experiment with three‐month‐old female ADLP^Tau^ mice before the known onset of pathology in order to test whether tau pathology could be exacerbated or prevented by manipulating NE levels. For the control group, we performed daily IP injections of PBS. Following previous studies (Andreasen et al., 2011; Chalermpalanupap et al., 2017; Ross & Stenfors, 2014; Wong et al., 2000), we injected RBX daily in one test group, while the other test group received DSP4 injections on specific days and PBS injections on the remaining days. Since it was sufficient to inject DSP4 once a month to decrease or maintain a low NE level in the brain (Chalermpalanupap et al., 2017; Ross & Stenfors, 2014), we administered the DSP4 injection once a month after the first two injections within a week (Figure 1a). We monitored the weights of all groups, including wild‐type mice without any injections, at the beginning, middle, and end of the injection period and found similar weights among them (Figure S2A). After two months of daily injections, we conducted behavioral tests. Initially, we carried out an open field test to assess locomotion in the groups, analyzing movements with EthoVision XT software, and observed similar locomotion values (Figure S2B–D). Then, we did three cognitive behavioral tests: Y‐maze, novel object recognition, and contextual fear conditioning tests. During the learning phases of these tests, the locomotion in all groups remained consistent (Figure S2E–G). Based on the observations, no mice displayed any sick signs or symptoms of illness. However, it was intriguing to note that only the mice given RBX exhibited cognitive deficits in all cognitive tests (Figure 1b–d).

### 3.3 RBX accelerates tau aggregation in the locus coeruleus of ADLP^Tau^ mice

**FIGURE 2:**
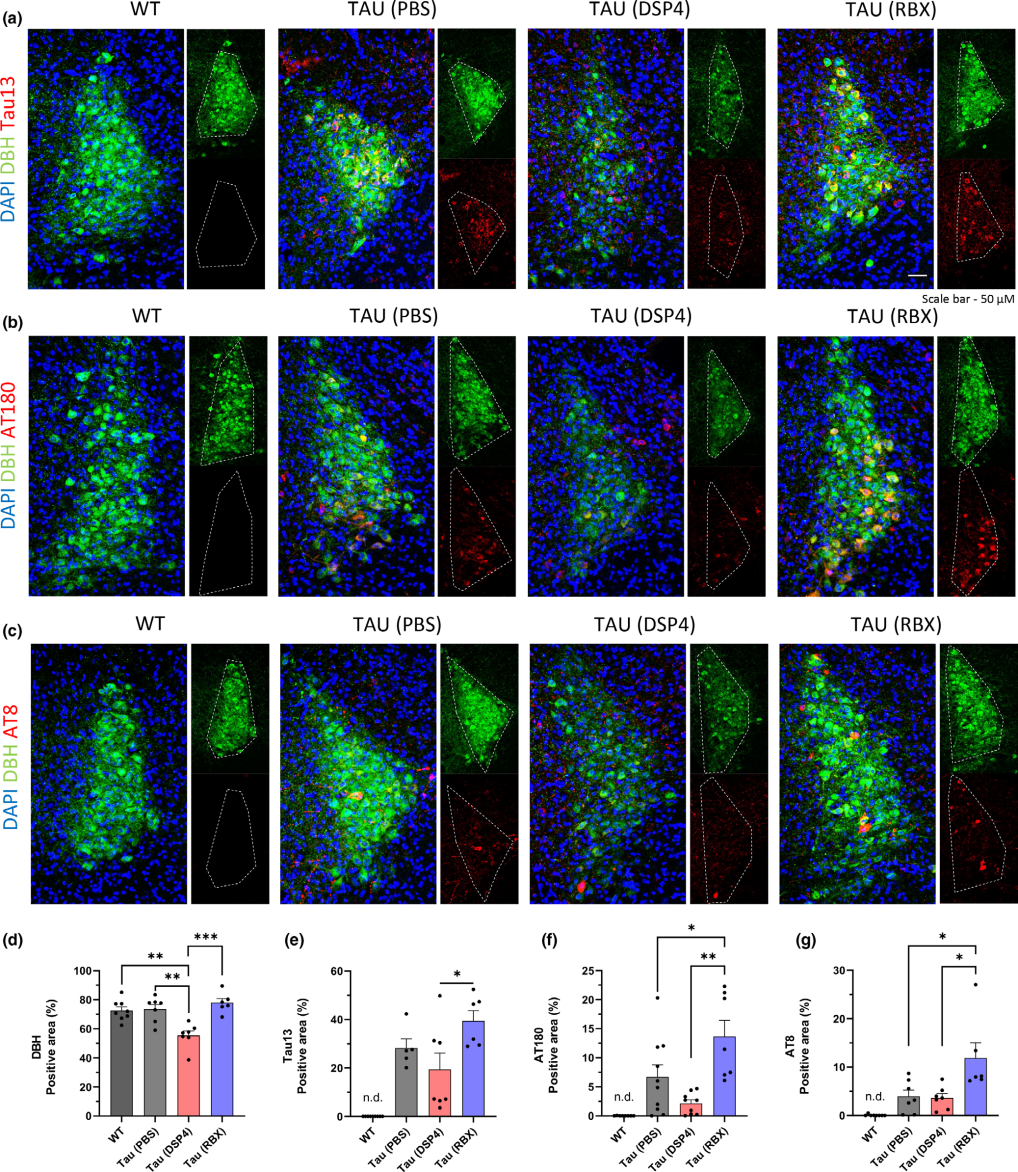
RBX exacerbates tau aggregation in the LC of ADLP^Tau^ mice. (a–c) Representative immunofluorescence images. (d–g) Quantification of DBH, Tau13, AT180, or AT8 positive area proportion in the LC following treatments in the ADLP^Tau^ mice. One dot in the bar graphs represents each mouse (d–g). All data represent mean ± SEM. Significance was determined by one‐way ANOVA and denoted by *p < 0.05, **p < 0.01, ***p < 0.001, or ****p < 0.0001.

After the behavioral tests, we sacrificed mice and collected brains. We used a hemisphere of each brain for immunohistochemistry (IHC) and the other hemisphere for western blotting. Because the LC primarily consists of medium‐size noradrenergic neurons and the drugs act through the neurons (Ross & Stenfors, 2014; Wong et al., 2000), we first examined tau pathology in the LC. By performing IHC, we observed that the mice given RBX obtained significantly more total human tau (Tau13) and hyperphosphorylated tau (AT180 and AT8) in the LC which could be identified by the noradrenergic neuronal marker (DBH) (Figure 2). We also confirmed that DSP4 induced neurodegeneration of the adrenergic neurons in the LC since the DBH signals of the mice with DSP4 were lowered in the LC (Figure 2d).

### 3.4 RBX also promotes tau aggregation in the hippocampus of ADLP^Tau^ mice

**FIGURE 3:**
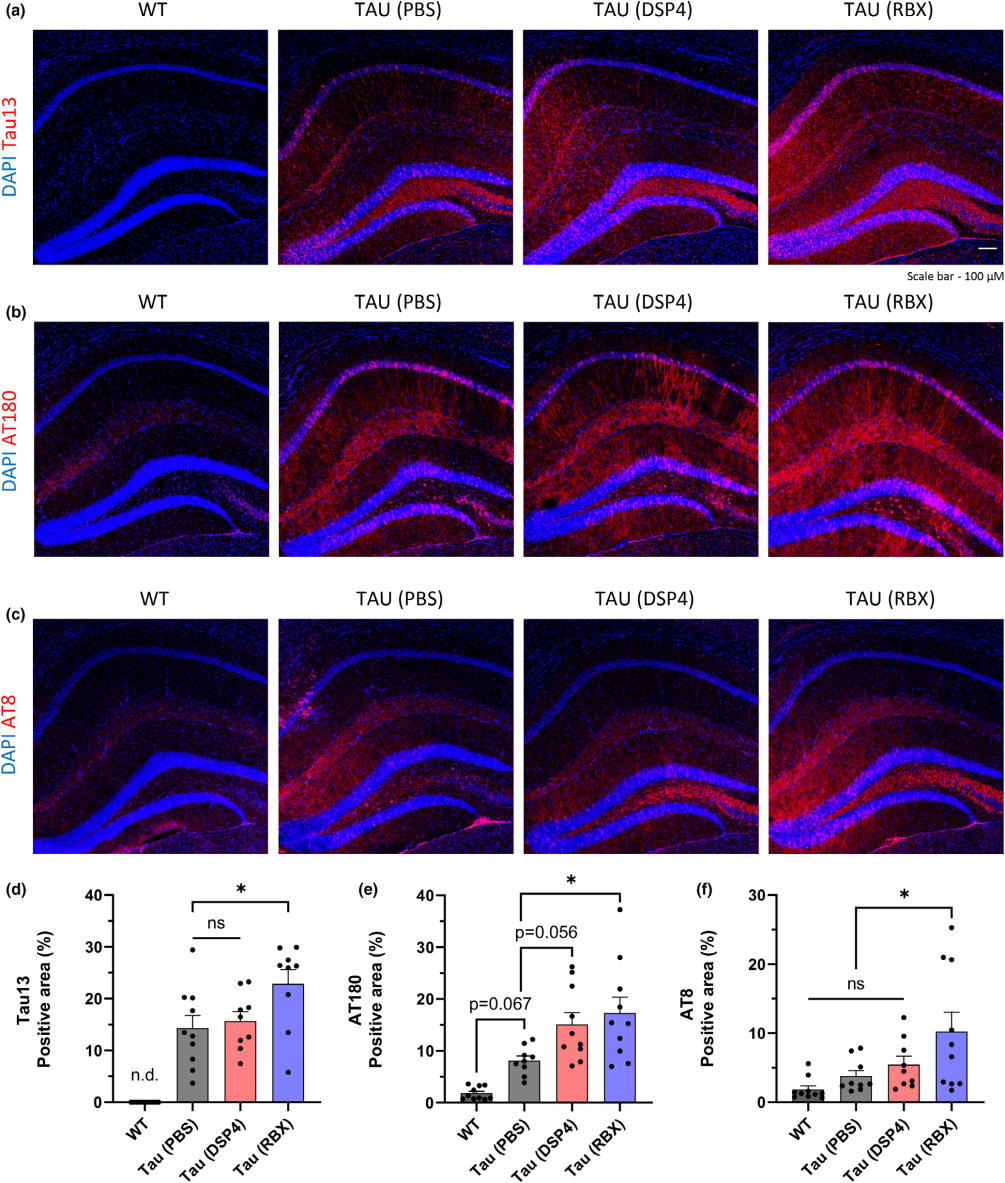
RBX also accelerates tau aggregation in the hippocampus of ADLP^Tau^ mice. (a–c) Representative immunofluorescence images. (d–f) Quantification of Tau13, AT180, or AT8 positive area proportion in the hippocampus following treatments in the ADLP^Tau^ mice. One dot in the bar graphs represents each mouse (d–f). All data represent mean ± SEM. Significance was determined by one‐way ANOVA and denoted by *p < 0.05, **p < 0.01, ***p < 0.001, or ****p < 0.0001.

By performing IHC, we found that the mice treated with RBX got significantly increased total human tau (Tau13) and hyperphosphorylated tau (AT180 and AT8) in the hippocampus in comparison to the other groups (Figure 3). In order to test whether these results could be reproduced, we isolated the hippocampus from the other side hemisphere and analyzed protein levels of Tau13, AT180, and AT8 through western blotting. We verified that the western blotting produced equivalent results (Figure S3). Furthermore, we demonstrated that the RBX‐induced rise in phosphorylated tau promoted the formation of tau aggregates by performing Congo Red staining. Congo Red intercalates and binds between the beta‐sheet structures of aggregated proteins (Frid et al., 2007). Notably, we detected a significant Congo Red signal in the hilus of the dentate gyrus, closely resembling the staining pattern of the AT8 antibody (Figure S4A–E). Subsequently, we conducted Pearson correlation analysis using both AT8 and Congo Red staining and observed a strong positive correlation between the two signals (Figure S4F). Collectively, these data indicate that the mice given RBX exhibited a significant increase in tau aggregation within the hippocampus as well as the LC.

### 3.5 Neurodegeneration arises in the hippocampal CA1 of the ADLP^Tau^ mice with RBX

**FIGURE 4:**
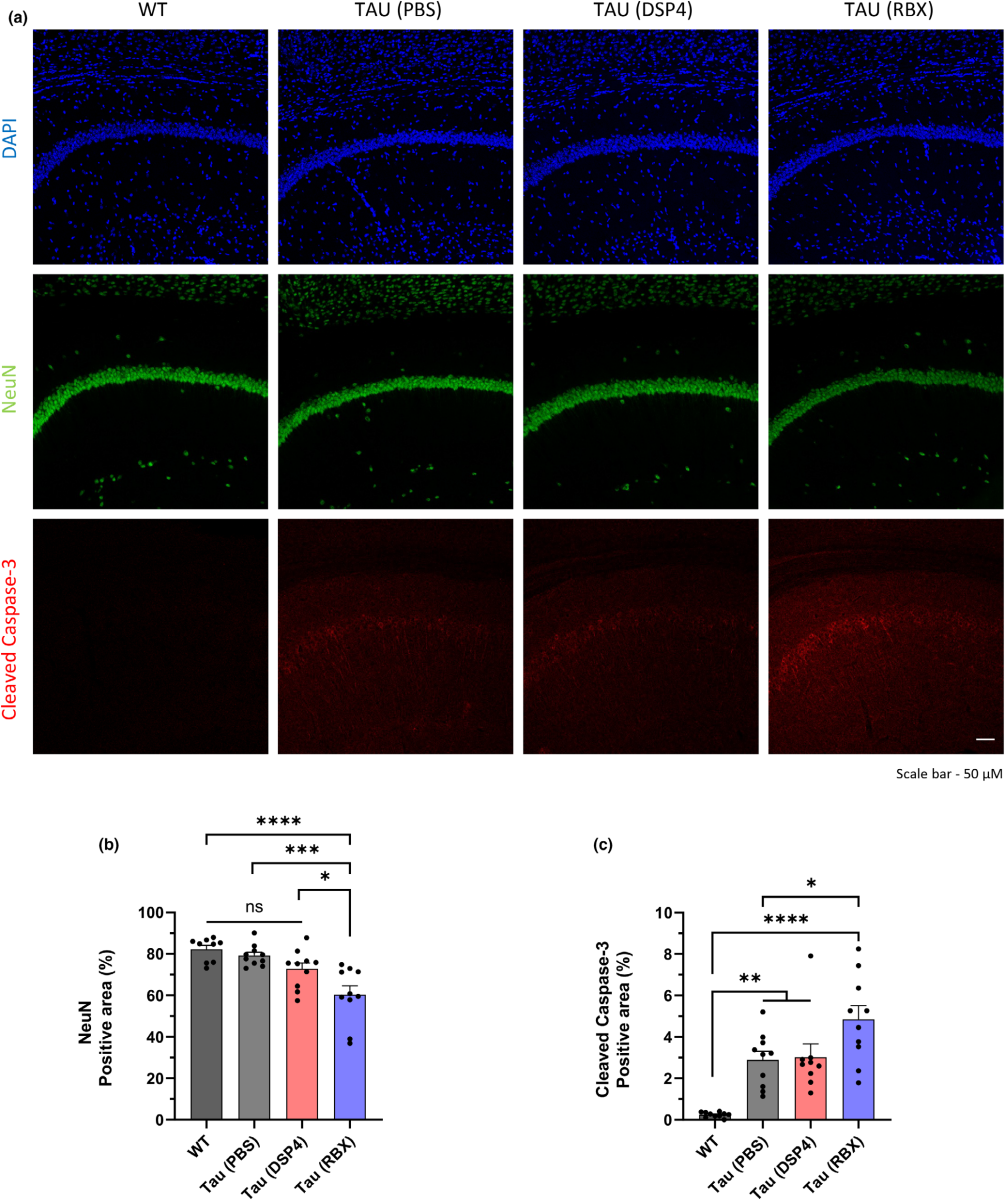
Apoptosis‐induced neuronal death occurs in the hippocampal CA1 of ADLP^Tau^ mice with RBX. (a) Representative immunofluorescence images. (b, c) Quantification of NeuN or cleaved caspase‐3 immunostaining in the hippocampal CA1 following treatments in the ADLP^Tau^ mice. One dot in the bar graphs represents each mouse (b, c). All data represent mean ± SEM. Significance was determined by one‐way ANOVA and denoted by *p < 0.05, **p < 0.01, ***p < 0.001, or ****p < 0.0001.

Because tau aggregation was known to lead to neuronal loss (Gao et al., 2018), we investigated whether neuronal cell deaths occurred in the hippocampus of the mice with tauopathy. We used NeuN antibody to apply fluorescence to neuronal nuclei and found that the signals of the fluorescence were diminished in the hippocampal CA1 of the mice injected with RBX (Figure 4a,b). In order to examine whether the cellular loss took place via apoptosis, we applied cleaved caspase‐3 antibody which binds active caspase‐3 enzyme known to play a key role in apoptosis (Porter & Jänicke, 1999). We observed that the signals of cleaved caspase‐3 were increased in the hippocampal CA1 of the mice with RBX (Figure 4a,c). In addition, we assessed whether the increase in tau deposition after RBX injection corresponded to the loss of synaptic integrity near the hippocampal CA1. We confirmed that the synaptic loss occurred in the mice treated with RBX as well as the neuronal loss (Figure S5). Based on the data, we propose that the RBX mice having tau pathology might experience neurodegeneration in the hippocampal CA1 caused by apoptosis.

### 3.6 Neither microgliosis nor astrogliosis affects tau pathology in the ADLP^Tau^ mice with RBX and vice versa

Next, we examined whether glial cells were associated with the tau pathology in the mice given RBX. We observed that glial cells had no impact on the tau pathology and vice versa, as determined by IHC. Our analysis revealed no signs of microgliosis or astrogliosis in both test groups administered with either DSP4 or RBX (Figure S6). According to the results, we discovered that the changes in NE levels induced by the drugs appeared to predominantly affect neurons in the brains.

### 3.7 Dopamine beta‐hydroxylase levels increase in the hippocampus of the ADLP^Tau^ mice with RBX

Since DSP4 is known as a selective neurotoxin to noradrenergic neurons, one of the representative enzymes in these neurons, dopamine beta‐hydroxylase (DBH), is diminished as a result of neuronal deaths caused by the drug (Chalermpalanupap et al., 2017; Ross & Stenfors, 2014). To assess DSP4's effectiveness in the brain, we conducted both IHC and western blot analyses to explore the protein levels of DBH. Our findings confirmed that DSP4 destroyed noradrenergic neurons, leading to NE depletion in the hippocampus as well as the LC (Figure 2d, Figure S7). When we evaluated DBH levels in the mice with DSP4, we also compared them to those in other groups. Interestingly, we discovered that DBH levels were elevated in the hippocampus of RBX‐treated mice with the highest levels of total human tau and hyperphosphorylated tau, compared to the same mice but injected with PBS (Figure S7). Furthermore, the tau transgenic mice with PBS showed higher DBH levels than the wild‐type mice (Figure S7). These findings reveal a positive correlation between tau pathology and DBH levels in the hippocampus.

### 3.8 RBX activates tau kinases, PKA, and GSK3β

**FIGURE 5:**
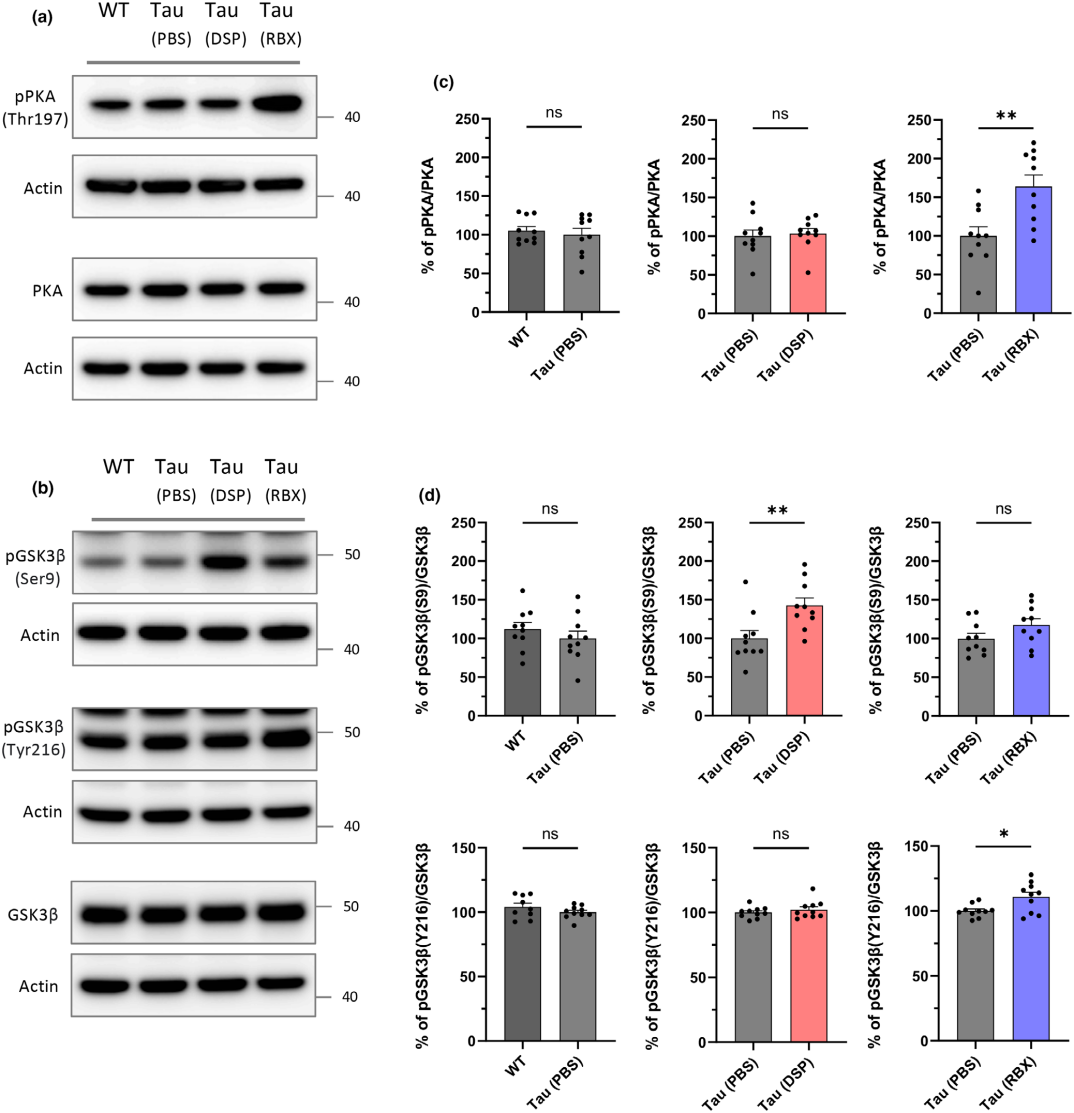
RBX facilitates the activities of tau kinases, PKA and GSK3β. (a, b) Representative western blot images. (c, d) Quantification of specific antibodies against pPKA, PKA, pGSK3β (S9), pGSK3β (Y216), or GSK3β in the hippocampus following treatments in the ADLP^Tau^ mice. One dot in the bar graphs represents each mouse (c, d). All data represent mean ± SEM. Significance was determined by unpaired *t* test and denoted by *p < 0.05, **p < 0.01, ***p < 0.001, or ****p < 0.0001.

As tau kinases phosphorylate tau proteins, leading to the aggregation of hyperphosphorylated tau into neurofibrillary tangles (NFTs) (Martin, Latypova, Wilson, Magnaudeix, Perrin, Yardin, & Terro, 2013), we sought to determine which tau kinases RBX activated. To evaluate their activity, we compared the protein levels of phosphorylated forms to total forms via western blotting. We selected eight well‐known tau kinases and identified that protein kinase A (PKA) and glycogen synthase kinase 3 beta (GSK3β) enzymes got overactivated by RBX among the candidates (Figure 5, Figure S8). The activation of PKA can be regulated through the activation loop of the catalytic subunit, specifically phosphorylation of threonine 197 (Thr197) (Adams et al., 1995). Similarly, GSK3β activity can either increase or decrease depending on the phosphorylation site. Phosphorylation at tyrosine 216 (Tyr216) enhances GSK3β activity, while phosphorylation at serine 9 (Ser9) inhibits it (Cole et al., 2004; Stambolic & Woodgett, 1994). In contrast to RBX, DSP4 reduced GSK3β activity by inducing phosphorylation at the Ser9 site (Figure 5b,d). Although both PKA and GSK3β showed increased activity by RBX, the upregulation of GSK3β was less pronounced. To confirm that GSK3β was overactivated by NE, we performed an in vitro assay. HEK293FT cells were transfected with plasmids encoding either GSK3β WT, GSK3β S9A, or GSK3β K85A and then treated with NE to examine whether NE induced phosphorylation at the active site (Tyr216) of GSK3β. As expected, cells expressing GSK3β K85A, a mutant that prevents phosphorylation at the active site (Cole et al., 2004), showed no increase in phosphorylation (Figure S9A,D). In contrast, cells expressing GSK3β WT or GSK3β S9A displayed increased phosphorylation at the active site following NE treatment (Figure S9A–C). Having identified PKA and GSK3β as hyperactivated kinases in response to RBX, we checked that these kinases substantially phosphorylated tau proteins. Specifically, tau phosphorylation sites associated with GSK3β, detected by the AT8 (Ser202, Thr205) and AT180 (Thr231) antibodies, were significantly elevated in the RBX group (Figures 2 and 3, Figures S3 and S4). Next, we investigated whether tau phosphorylation sites associated with PKA (Ser262, Ser409) were similarly increased in the RBX group (Ko et al., 2019). Our results indicated that tau mice treated with RBX exhibited the highest phosphorylation levels at Ser262 and Ser409, whereas mice treated with DSP4 showed reduced phosphorylation at these sites compared to PBS‐treated controls (Figure S10). This suggests that elevated NE levels enhance PKA activity, leading to increased tau phosphorylation, while reduced NE levels lower PKA activity and decrease tau phosphorylation.

### 3.9 Both DSP4 and RBX reduce PP2A activity

Following our investigation of tau kinase activity in relation to the aggregation of hyperphosphorylated tau in ADLP^Tau^ mice treated with RBX, we extended our analysis to protein phosphatases that could dephosphorylate tau (Martin, Latypova, Wilson, Magnaudeix, Perrin, & Terro, 2013). We focused on two key phosphatases, PP1 and PP2A, and found that PP2A activity was significantly reduced in both the DSP4‐ and RBX‐treated groups, as indicated by the quantification of phosphorylated/total PP2A levels (Figure S11). In other words, long‐term alterations in NE levels in both directions, induced by the drugs, could inhibit the activation of PP2A via phosphorylation at tyrosine 307, potentially contributing to the accumulation of hyperphosphorylated tau.

### 3.10 Elevated NE level also accelerates tau aggregation in the human brain organoid

**FIGURE 6:**
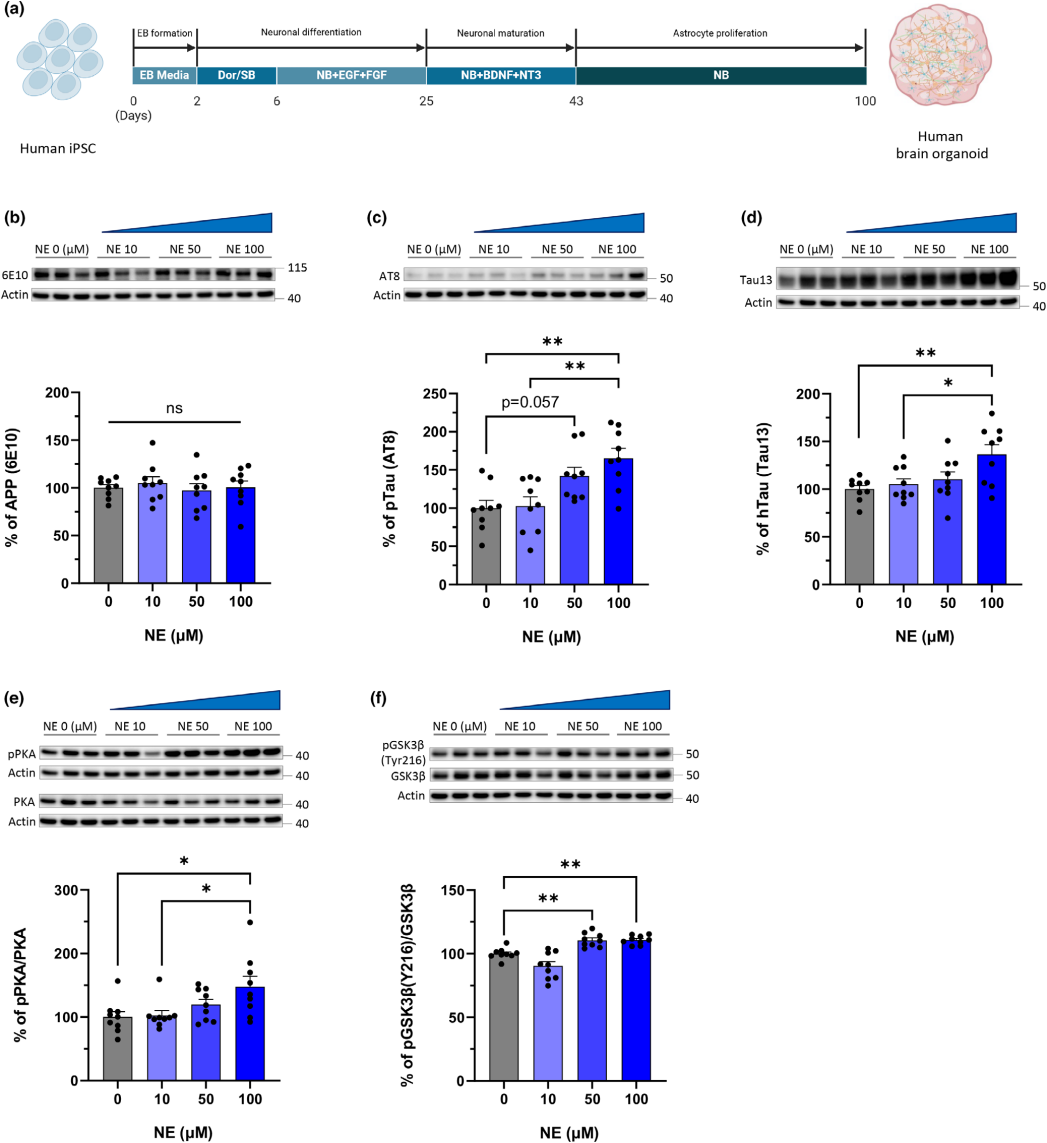
Human brain organoids exposed to the elevated NE facilitate the activities of PKA and GSK3β and aggravate tau aggregation. (a) Schematic representation of the experimental design for generating human brain organoids. (b–f) Representative western blot images and quantification of specific antibodies against 6E10, AT8, Tau13, pPKA, PKA, pGSK3β (Y216), or GSK3β in the human brain organoids exposed to various levels of NE. One dot in the bar graphs represents each organoid (b–f). All data represent mean ± SEM. Significance was determined by one‐way ANOVA and denoted by *p < 0.05, **p < 0.01, ***p < 0.001, or ****p < 0.0001.

To ensure that the data relating NE and tauopathy in humans matched our murine findings, we created human brain organoids from human‐induced pluripotent stem cells (hiPSCs) (Figure 6a). We selected the BIONi010‐C‐2 (APOE3/3) cell line to detect early changes in Alzheimer's disease (AD) pathology markers, such as amyloid beta or tau aggregation, upon exposure of the matured organoids to increased norepinephrine (NE). This cell line originated from fibroblasts of a healthy male African–American, and we expected it to exhibit significantly lower baseline levels of AD pathology markers compared to those derived from AD patients. First, we confirmed the maturity of the brain organoids through IHC (Figure S12). Following established protocols (Park et al., 2021), these organoids were classified as cortical brain organoids, which typically lack NE‐producing cells primarily found in the brainstem. Therefore, to replicate drug‐mediated regulation of NE levels observed in mouse brains, we directly administered NE to the cortical brain organoids. This was necessary because the drugs used in mouse experiments modulate NE levels by targeting NE‐producing cells. Western blot analysis revealed that while amyloid beta precursor protein (APP) levels remained unchanged, there was a positive correlation between NE exposure and the levels of phosphorylated tau and total human tau (Figure 6b–d). Furthermore, our findings demonstrated a link between NE and the activation of tau kinases, specifically PKA and GSK3β, consistent with observations from our mouse model (Figure 6e,f). Thus, we propose that overactivation of kinases by elevated NE levels might exacerbate tau aggregation in both human brain organoids and mouse brains.

## 4 DISCUSSION

Neurotransmitter involvement in Alzheimer's disease (AD) has long been studied (Kandimalla & Reddy, 2017). Norepinephrine (NE), a neurotransmitter implicated in a variety of cognitive tasks including alertness, motivation, learning, and memory (Benarroch, 2009), has received particular attention. The locus coeruleus (LC), which is primarily responsible for NE production and distribution throughout the brain, is susceptible to high amounts of NE and pathogenic forms of tau (Braak et al., 2011; Theofilas et al., 2016). Later, tau accumulation causes LC neurons to degenerate, resulting in a decrease in NE levels and cognitive deficits (Braak et al., 2011; Theofilas et al., 2016). This pattern has sparked research into the potential function of NE in disease etiology. The purpose of this study was to explore the relationship between NE and tau pathology in AD. For the murine data, we used two different drugs, N‐(2‐chloroethyl)‐N‐ethyl‐2‐bromobenzylamine (DSP4) and reboxetine (RBX), to manipulate NE levels in the brains of ADLP^Tau^ mice. To examine the efficacy of these drugs in pathogenesis, we initiated treatments in early tau mice before the known onset of tau pathology. We found that prolonged exposure to increased NE in the brain, induced by the antidepressant RBX, aggravated tau aggregation, neuronal death, and cognitive impairments in ADLP^Tau^ mice. In our investigation of human data, we generated human brain organoids and applied various levels of NE to them. Surprisingly, our findings revealed that heightened NE exposure in the organoids accelerated tau aggregation, mirroring the results obtained from the murine data.

Several conclusions can be drawn from the data analysis. To begin, our findings demonstrated that DSP4 had no significant effect on tau pathology in tau transgenic mice. In both immunohistochemical (IHC) and western blot examinations, the DSP4 group only revealed a substantial increase in hyperphosphorylation at the threonine (Thr) 231 location of the tau protein (AT180) compared to the PBS control group (Figure 3, Figure S3). Given that hyperphosphorylation at the Thr231 location is identified as an early occurrence of tau pathology in tau transgenic mice (Augustinack et al., 2001), we suggest that DSP4 may also contribute to the acceleration of tau pathology. The progression of tau pathology in the DSP4 group, however, may be slower than in the RBX group. Only the RBX group exhibited significantly increased hyperphosphorylation at the serine (Ser) 202/Thr205 sites of the tau protein (AT8) (Figure 3, Figure S3), in addition to the Thr231 site, indicating a middle to late stage of tau pathology (Augustinack et al., 2001). As a result, during the two‐month injection period of this young tau animal experiment, the DSP4 group did not exhibit any symptoms or signs induced by hyperphosphorylated tau, such as neuronal death, synapse loss, or cognitive dysfunction (Figures 1 and 4, Figure S5). It is probable that if the injection period were extended, the DSP4 group might eventually show comparable characteristics to the RBX group. Previous research has shown that administering DSP4 to P301S tau transgenic mice for 4 or 6 months worsened tau pathology, including tau aggregation, neuronal loss, and cognitive impairments (Chalermpalanupap et al., 2017).

Second, while our findings demonstrated that RBX‐induced tau aggregation occurred in both the LC and the hippocampus, we were unable to show that the tau aggregation caused neurodegeneration in the LC. To observe neurodegeneration in the LC, we utilized an antibody against dopamine beta‐hydroxylase (DBH), one of the noradrenergic neuronal markers. Surprisingly, DBH levels were not lower in the RBX group than in the PBS control group in both IHC and western blotting, despite the fact that the RBX group experienced considerable tau aggregation (Figure 2d, Figure S7). According to previous research (Giubilei et al., 2004), we suggest that the alterations in DBH might represent subsequent compensation for the loss of noradrenergic neurons and/or the blockage of NE reuptake transporters on the presynaptic membrane of neurons by RBX to maintain intra‐ or extracellular levels of NE. In contrast, DSP4 showed significantly lower DBH levels in the LC and hippocampus in both IHC and western blotting (Figure 2d, Figure S7). Because DSP4 specifically targets noradrenergic neurons and eliminates a majority of these neurons that carry DBH and tau proteins, maintaining DBH levels through compensatory mechanisms may become challenging. Furthermore, due to the substantial neuronal loss, the DSP4 group seemed to have fewer hyperphosphorylated tau and tau aggregates in the LC than the other groups (Figure 2). In other words, we cannot claim that the drug restored tauopathy in the LC based on this phenomenon.

Third, we employed a cleaved caspase‐3 antibody to investigate whether neuronal loss was caused by apoptosis. We found higher cleaved caspase‐3 signals in the hippocampal CA1 region of RBX‐treated mice, displaying neuronal loss in the same location (Figure 4). We suggest that active caspase‐3 is involved in neurodegeneration caused by apoptosis. However, further research is needed to establish a direct relationship between caspase‐3 and neuronal loss in apoptosis. Caspase‐3 has previously been demonstrated to cleave tau proteins, inducing tau aggregation rather than serving an apoptotic role (Means et al., 2016). These aggregated tau forms can contribute to neuronal death via a variety of mechanisms, including apoptosis (Gao et al., 2018). The mechanisms that govern the interaction between tau and caspase‐3 are complicated and varied. In the near future, better knowledge of the link will be required to create new treatment strategies for AD.

Finally, after investigating tau pathology, we focused on prospective tau kinases to understand how RBX‐induced higher NE levels in the brain enhanced hyperphosphorylated tau formation. We determined that the only RBX group experienced overactivated protein kinase A (PKA) and glycogen synthase kinase 3 beta (GSK3β) among putative tau kinases when compared to the other groups (Figure 5, Figure S8). PKA is activated by NE‐induced increases in cellular cyclic‐AMP (cAMP) (Lodish, 2016). Then, PKA activation directly phosphorylates cyclic‐AMP response element‐binding protein (CREB), a transcription factor known for regulating neuronal growth, survival, differentiation, proliferation, synaptic plasticity, and more (Sharma & Singh, 2021). CREB may also suppress tau expression at both the mRNA and protein levels (Liu et al., 2015; Sharma & Singh, 2021). Therefore, NE depletion via DSP4 may result in reduced CREB activity, hastening tau aggregation. Despite the protective effects of the cAMP‐PKA‐CREB signaling pathway on tau pathology, PKA hyperactivated by RBX can disrupt recognition memory and spatial memory and accelerate tau phosphorylation (Benítez et al., 2021; Giralt et al., 2011). In addition, hyperactive PKA may even prime tau phosphorylation before GSK3β does (Liu et al., 2006). Our findings also imply that NE levels can influence GSK3β activity. Decreased NE caused by DSP4 reduced GSK3β activity through serine 9 (Ser9) phosphorylation (Figure 5b,d). Conversely, increased NE by RBX boosted GSK3β activity via tyrosine 216 (Tyr216) phosphorylation (Figure 5b,d). GSK3β is a critical factor in AD pathophysiology because its kinase activity rises with age and in AD pathology (Lauretti et al., 2020; Lee et al., 2006). GSK3β hyperactivity encourages the phosphorylation and the formation of toxic tau species (Lee et al., 2006). Because RBX overactivates both PKA and GSK3β associated with tau phosphorylation, rapid tauopathy might ensue despite the favorable effects of CREB activation on the disease. Based on our findings and previous studies, we suggest probable molecular mechanisms underlying tau aggregation at various levels of NE, as illustrated in the graphical figure.

In conclusion, it is crucial to carefully evaluate both the potential benefits and risks of modulating NE levels in the brain. Our findings suggest that both high and low NE levels can induce tau pathology. Maintaining optimal NE levels in the central nervous system is likely essential to harness the positive effects of NE, potentially preventing or delaying the onset of AD. However, further research is imperative to fully understand the complex relationship between NE and tau pathology in AD, especially in humans, in order to develop safe and effective therapeutics.

## AUTHOR CONTRIBUTIONS

J.‐H.J. and I.M.‐J. conceived and designed the study. D.K.K. and S.C. gave much advice on how to perform and analyze the experiments well such as western blot and immunohistochemistry. They also helped J.‐H.J. conduct behavioral tests. J.W.H. and J.H. provided matured human brain organoids and gave advice specifically how to perform the experiments related to the organoids. J.‐H.J. wrote the manuscript under the supervision of I.M.‐J.

## CONFLICT OF INTEREST STATEMENT

None declared.

## Supporting information


Figure S1‐S12.

## Data Availability

All data supporting this work are included in the publication and/or the Supplementary Materials. The source datasets are also available from the corresponding author upon request.
