# Supplementary material for: Long‐term exposure to excessive norepinephrine in the brain induces tau aggregation, neuronal death, and cognitive deficits in early tau transgenic mice
Source: Aging Cell. 2024 Nov 26;24(3):e14420. doi: 10.1111/acel.14420 (PMC11896411; doi:10.1111/acel.14420)
Supplement: Supplementary file 1 — Figure S1‐S12. [file ACEL-24-e14420-s001.pdf]

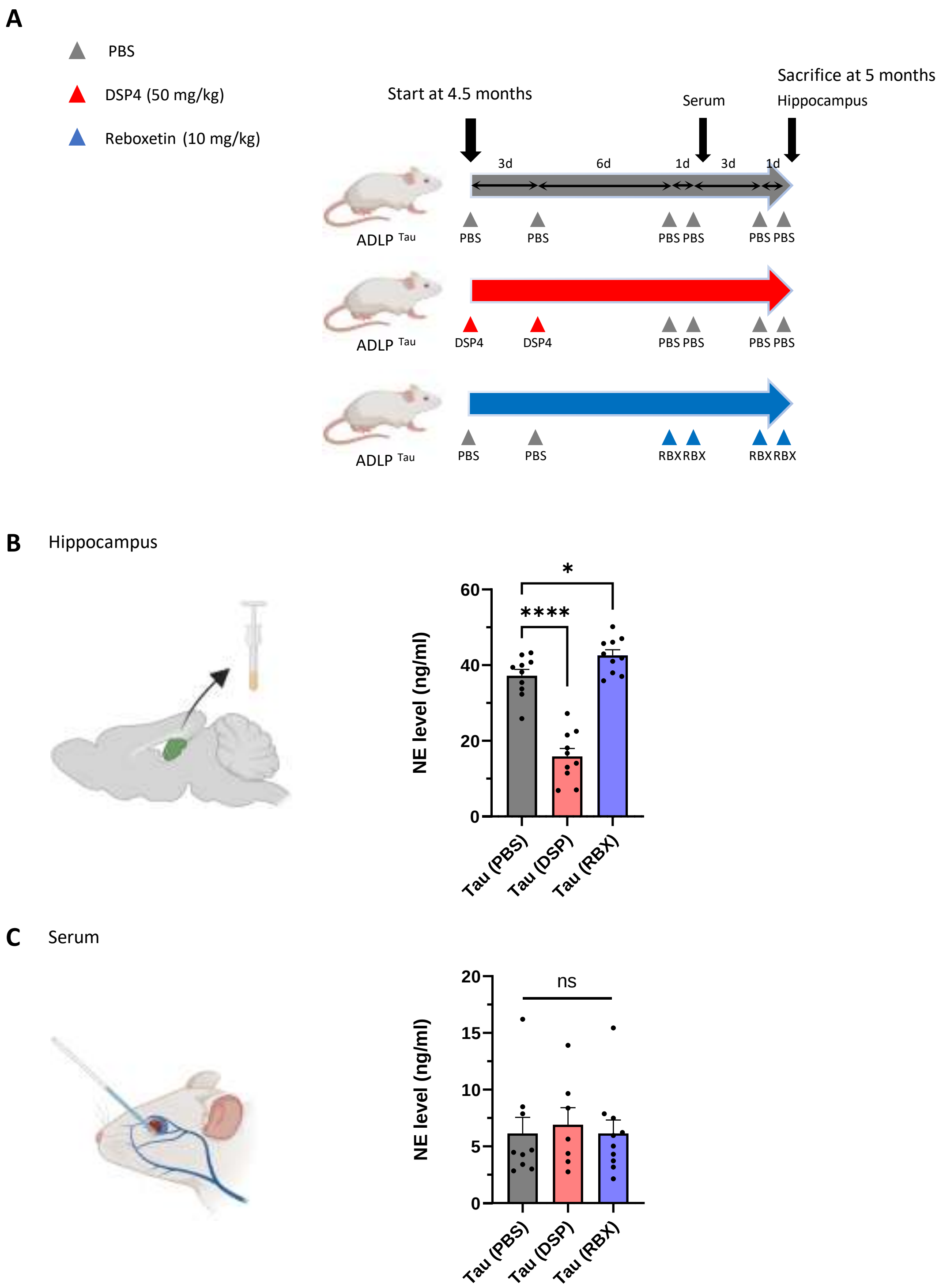

Supplementary Fig. 1

Both DSP4 and RBX can alter the NE levels in the hippocampus of ADLP^{Tau} mice. **A** Schematic representation of the experimental design. **B-C** Quantification of NE levels in the hippocampus or serum following the drug injections in the ADLP^{Tau} mice. One dot in the bar graphs represents each mouse (**B-C**). All data represent mean±SEM. Significance was determined by one-way ANOVA and was denoted by *p < 0.05, **p < 0.01, ***p < 0.001, or ****p < 0.0001.

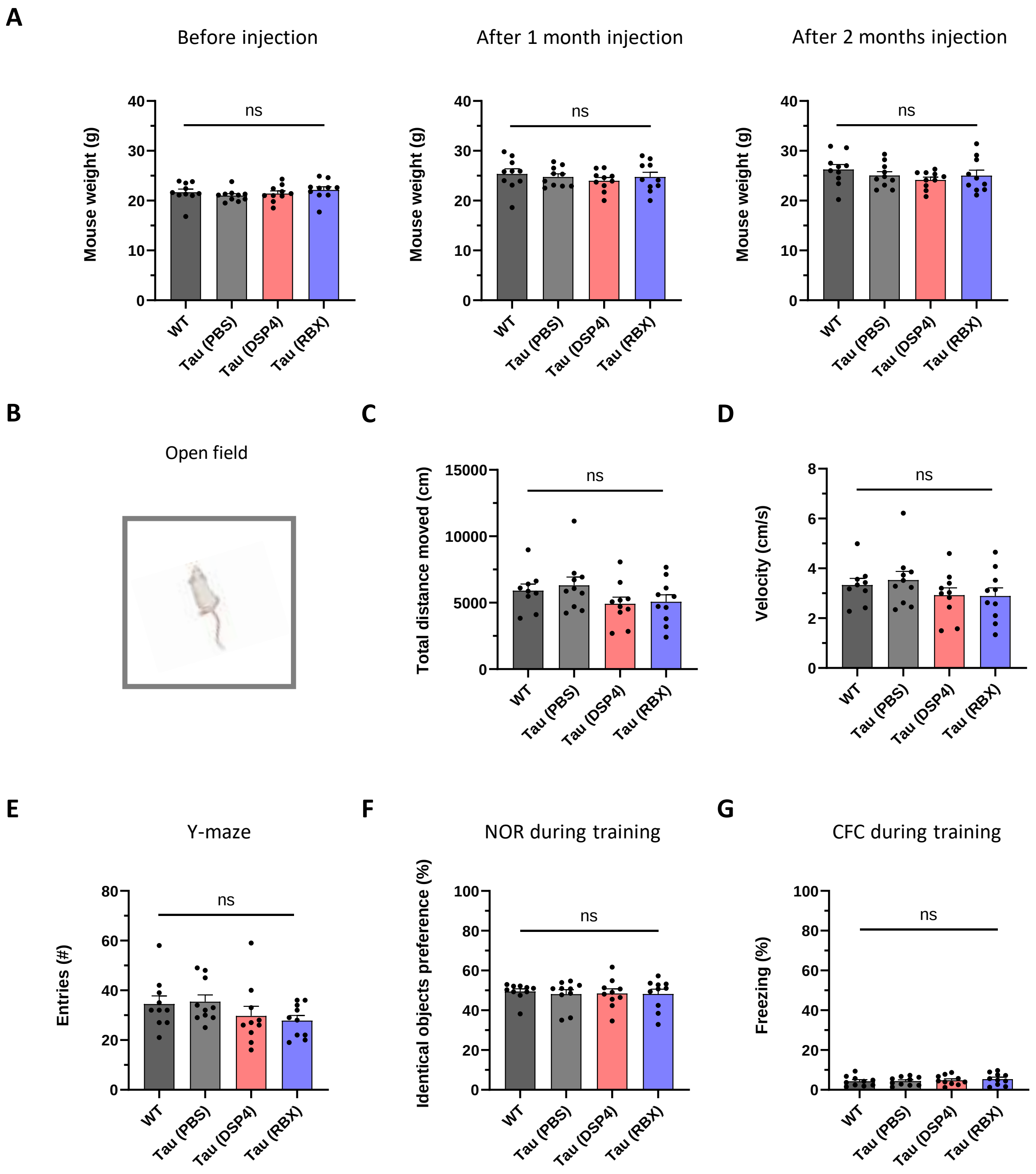

Supplementary Fig. 2

None of the ADLP^{Tau} mice exhibit any sick sign or symptom regardless of drugs. **A** Graphs of body weights of mice before and after treatments. **B** Representative open field test image. **C-D** Graphs of total distance moved and velocity in the open field test. **E** The number of arm entries in the Y-maze task. **F** Percentage of identical objects preference during training for the novel object recognition test. **G** Percentage of freezing during training in the contextual fear conditioning test. One dot in the bar graphs represents each mouse (**B-D**). All data represent mean±SEM. Significance was determined by one-way ANOVA and was denoted by *p < 0.05, **p < 0.01, ***p < 0.001, or ****p < 0.0001.

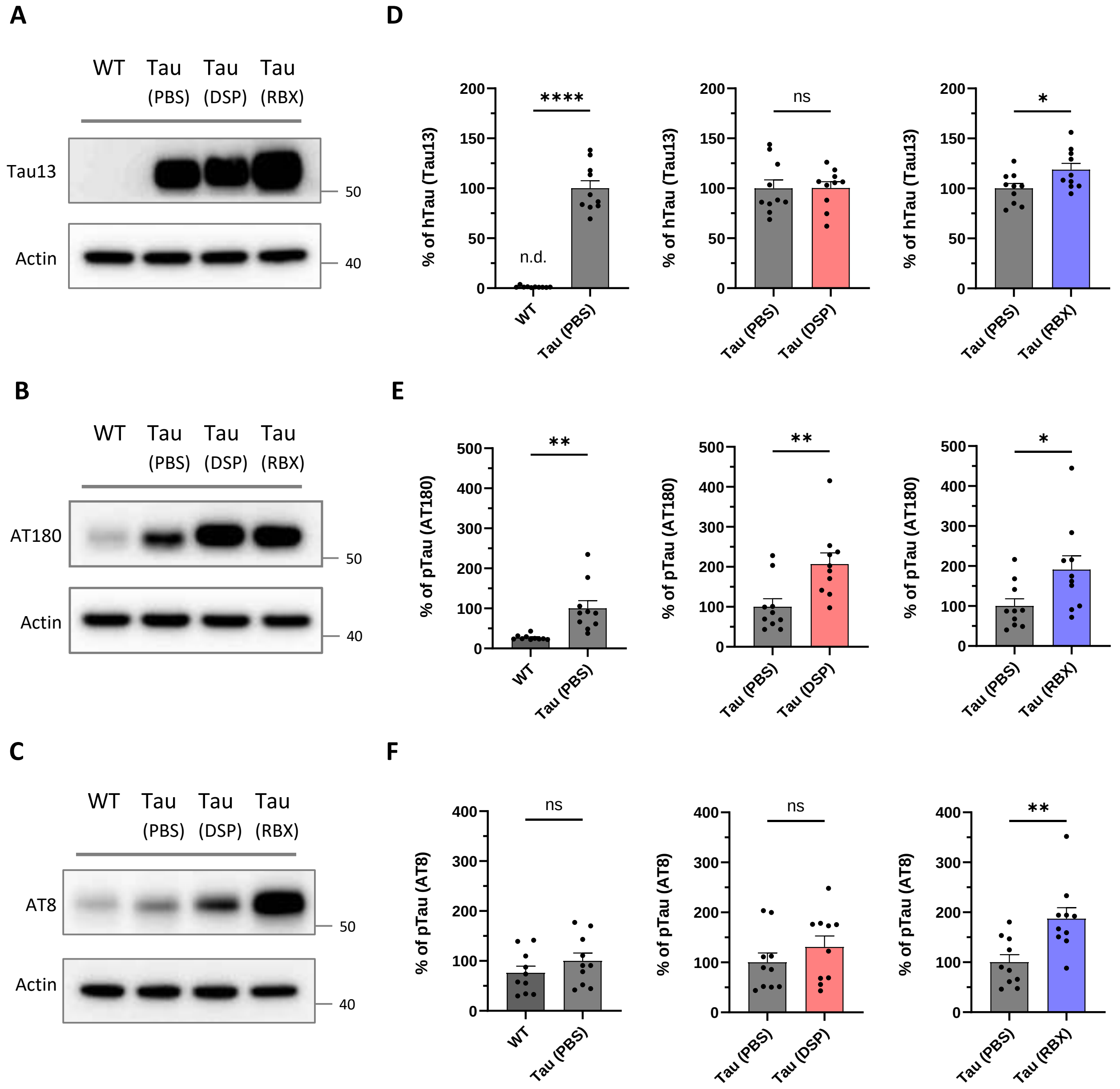

Supplementary Fig. 3

RBX hastens tau aggregation of ADLP^{Tau} mice in the western blot. **A-C** Representative western blot images. **D-F** Quantification of specific antibodies against Tau13, AT180, or AT8 in the hippocampus following treatments in the ADLP^{Tau} mice. One dot in the bar graphs represents each mouse (**D-F**). All data represent mean±SEM. Significance was determined by unpaired t-test and was denoted by *p < 0.05, **p < 0.01, ***p < 0.001, or ****p < 0.0001.

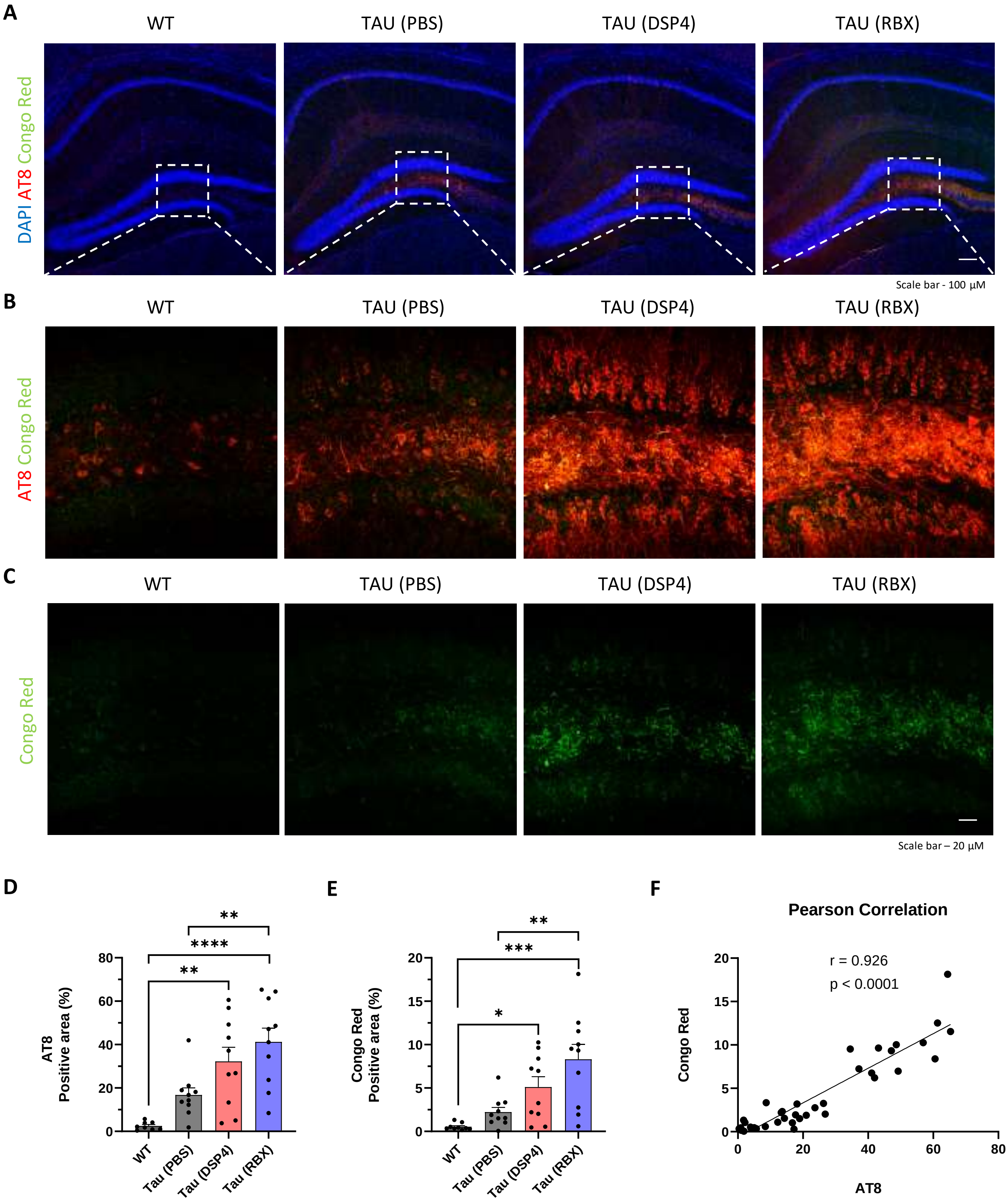

Supplementary Fig. 4

RBX-induced increase in phosphorylated tau facilitates the formation of tau aggregates. **A-C** Representative fluorescence images. **D-E** Quantification of AT8 or Congo Red positive area proportion in the hilus of the dentate gyrus following treatments in the ADLP^{Tau} mice. **F** Pearson correlation analysis between AT8 and Congo Red. One dot in the bar graphs represents each mouse (**D-F**). All data represent mean±SEM. Significance was determined by one-way ANOVA and was denoted by *p < 0.05, **p < 0.01, ***p < 0.001, or ****p < 0.0001.

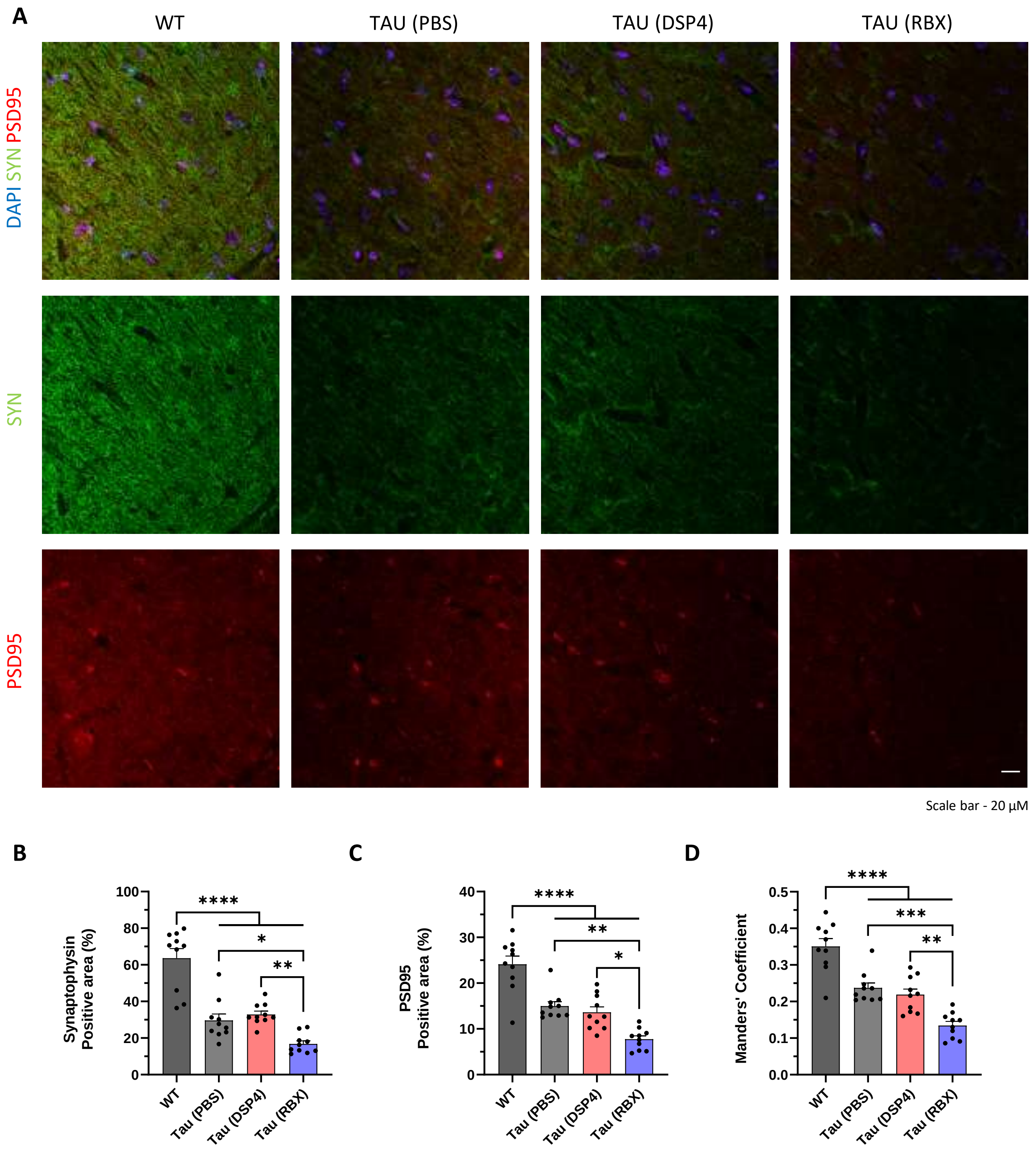

Supplementary Fig. 5

Synaptic loss accelerates in the hippocampal CA1 of ADLP^{Tau} mice with RBX. **A** Representative immunofluorescence images. **B-C** Quantification of specific antibodies against Synaptophysin or PSD95 in the hippocampal CA1 following treatments in the ADLP^{Tau} mice. **C** Colocalization quantification by Manders' coefficient (M1: fraction of Synaptophysin overlapping PSD95). One dot in the bar graphs represents each mouse (**B-D**). All data represent mean±SEM. Significance was determined by one-way ANOVA and was denoted by *$p < 0.05$, **$p < 0.01$, ***$p < 0.001$, or ****$p < 0.0001$.

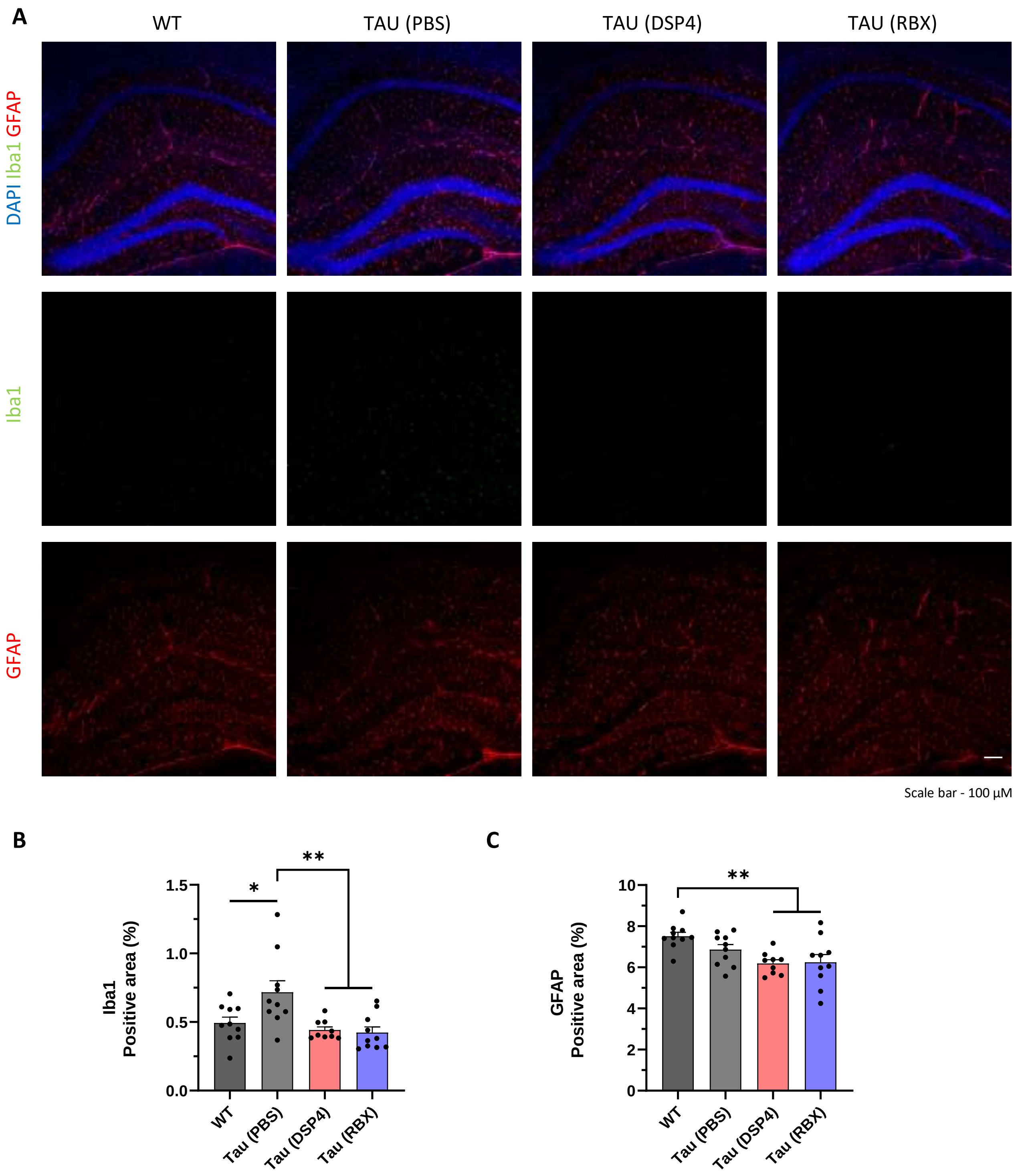

Supplementary Fig. 6

Microgliosis and astrogliosis have no effect on tau pathology in ADLP^{Tau} mice with RBX, and vice versa. **A** Representative immunofluorescence images. **B** Quantification of specific antibodies against Iba1 or GFAP in the hippocampus after treatments in the ADLP^{Tau} mice. One dot in the bar graphs comes from each mouse (**B**). All data represent mean±SEM. Significance was determined by one-way ANOVA and was denoted by $*p < 0.05$, $**p < 0.01$, $***p < 0.001$, or $****p < 0.0001$.

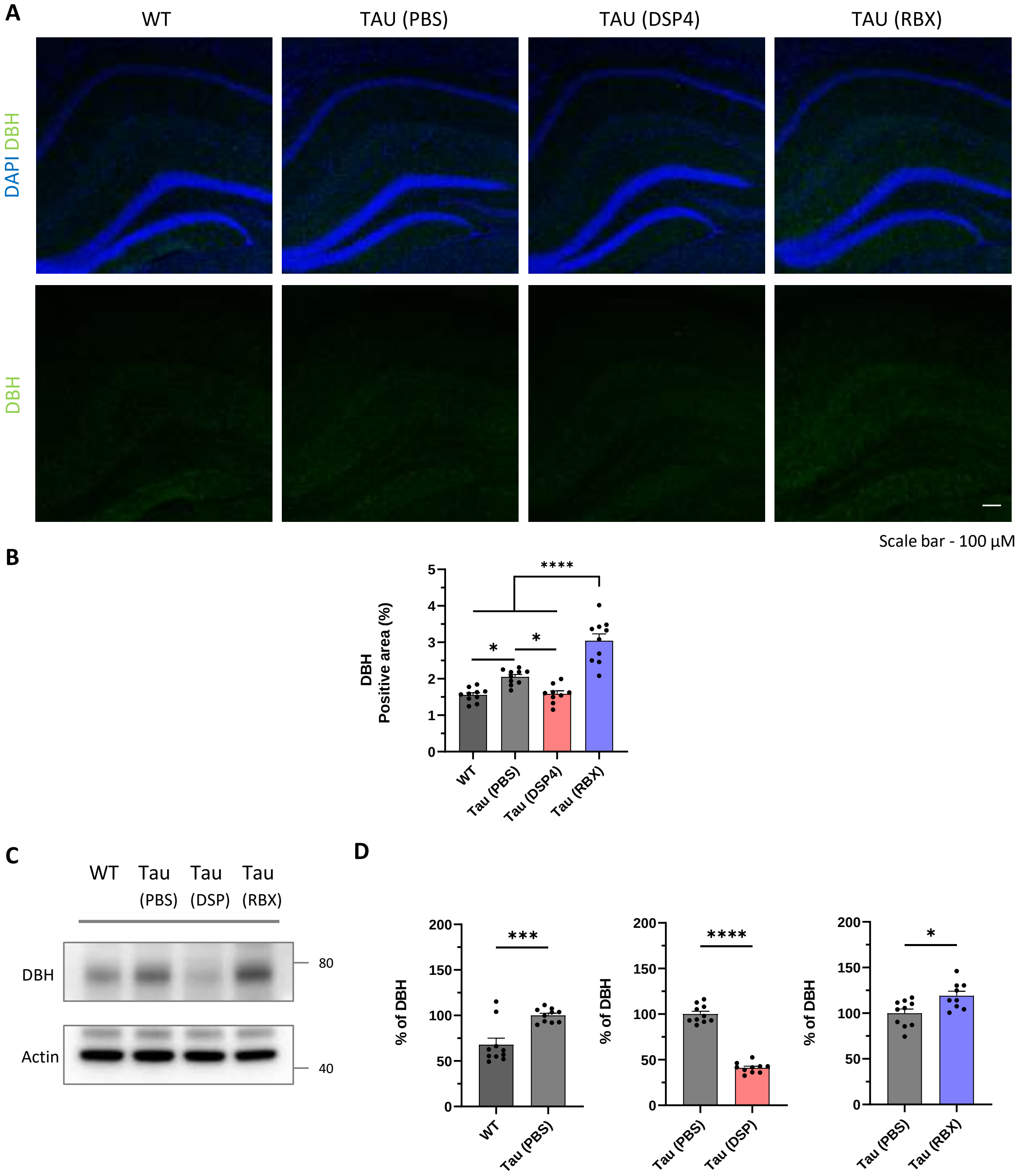

Supplementary Fig. 7

DBH increases in the hippocampus of ADLP^{Tau} mice with RBX. **A** Representative immunofluorescence images. **B** Quantification of DBH immunostainings in the hippocampus following treatments in the ADLP^{Tau} mice. **C** Representative western blot images. **D** Quantification of DBH immunoblots in the hippocampus following treatments in the ADLP^{Tau} mice. One dot in the bar graphs represents each mouse (**B, D**). All data represent mean±SEM. Significance was determined by one-way ANOVA **(B)** or unpaired t-test **(D)** and was denoted by *$p < 0.05$, **$p < 0.01$, ***$p < 0.001$, or ****$p < 0.0001$.

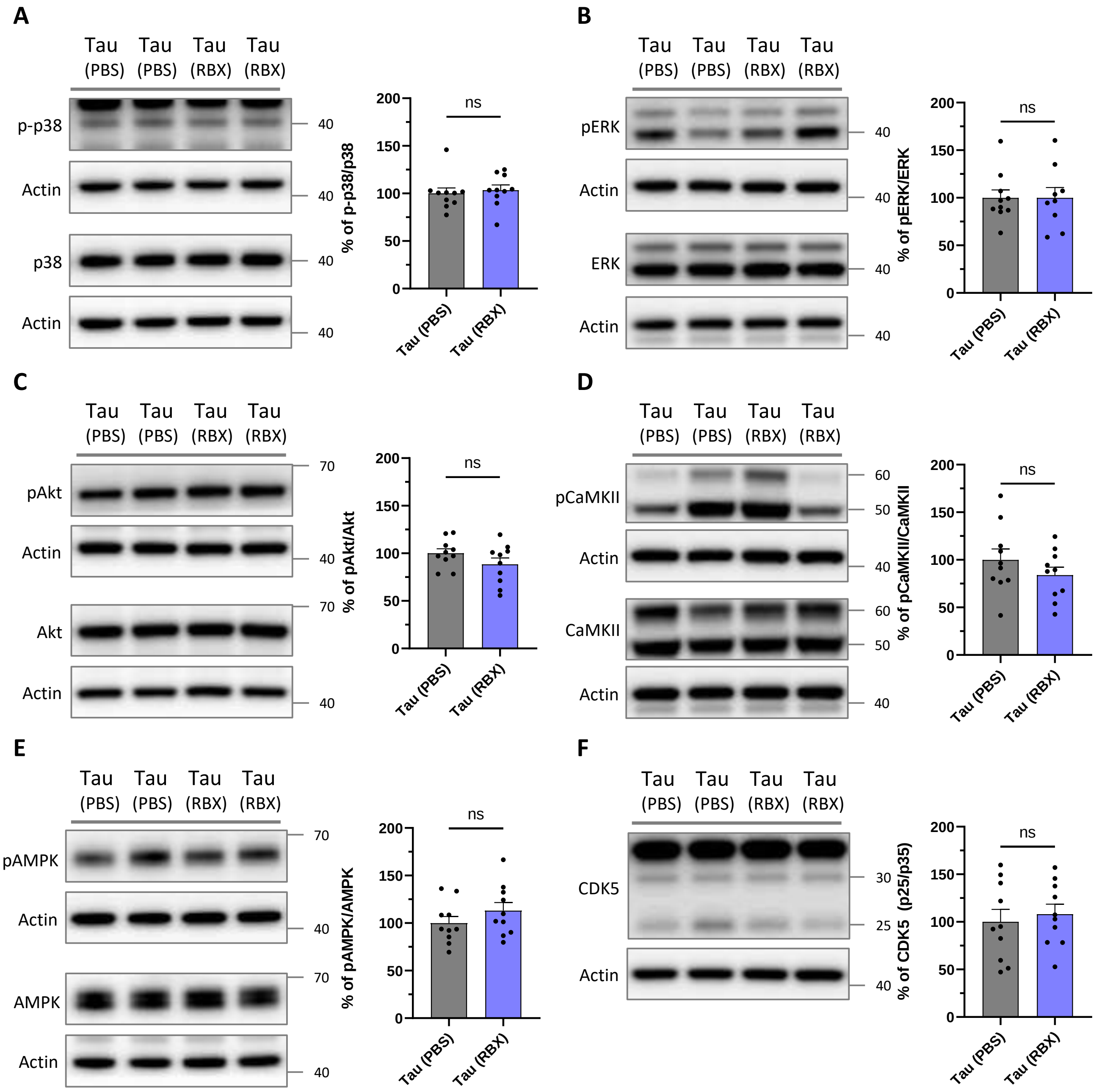

Supplementary Fig. 8

Six candidate tau kinases are independent of RBX. **A-F** Representative western blot images and quantification of specific antibodies against p-p38, p38, pERK, ERK, pAkt, Akt, pCaMKII, CaMKII, pAMPK, AMPK, or CDK5 in the hippocampus following treatments in the ADLP^{Tau} mice. One dot in the bar graphs represents each mouse (**A-F**). All data represent mean±SEM. Significance was determined by unpaired t-test and was denoted by *p < 0.05, **p < 0.01, ***p < 0.001, or ****p < 0.0001.

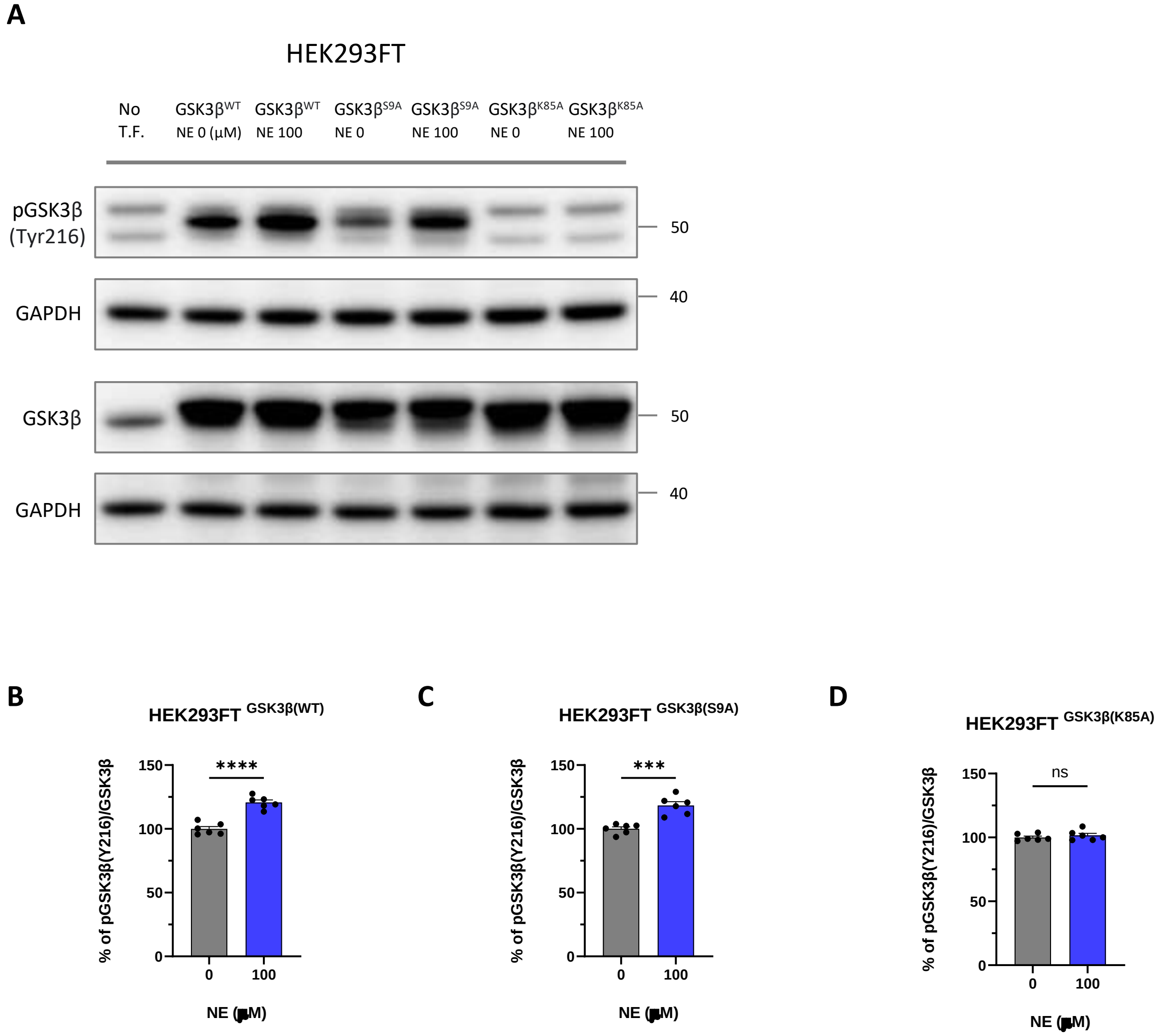

Supplementary Fig. 9

NE treatment increases phosphorylation at the active site of GSK3β in an in vitro assay. **A** Representative western blot image. **B-D** Quantification of specific antibodies against pGSK3β(Y216) or GSK3β in the transfected HEK293FT cells following NE treatment. One dot in the bar graphs comes from each culture well (**C-D**). All data represent mean±SEM. Significance was determined by unpaired t-test and was denoted by *p < 0.05, **p < 0.01, ***p < 0.001, or ****p < 0.0001.

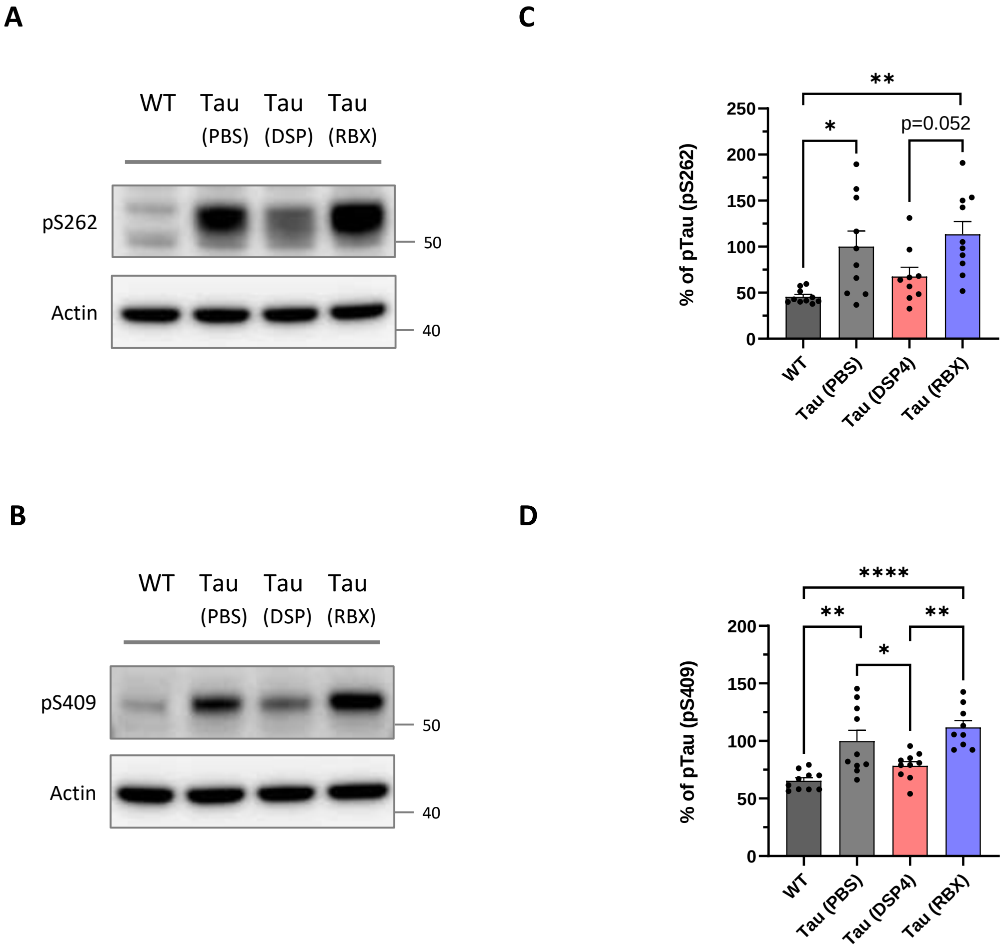

Supplementary Fig. 10

Both DSP4 and RBX affect the PKA-related phosphorylation of the tau protein. **A-B** Representative western blot images. **C-D** Quantification of specific antibodies against pS262 or pS409 in the hippocampus following treatments in the ADLP^{Tau} mice. One dot in the bar graphs represents each mouse (**C-D**). All data represent mean±SEM. Significance was determined by one-way ANOVA and was denoted by *p < 0.05, **p < 0.01, ***p < 0.001, or ****p < 0.0001.

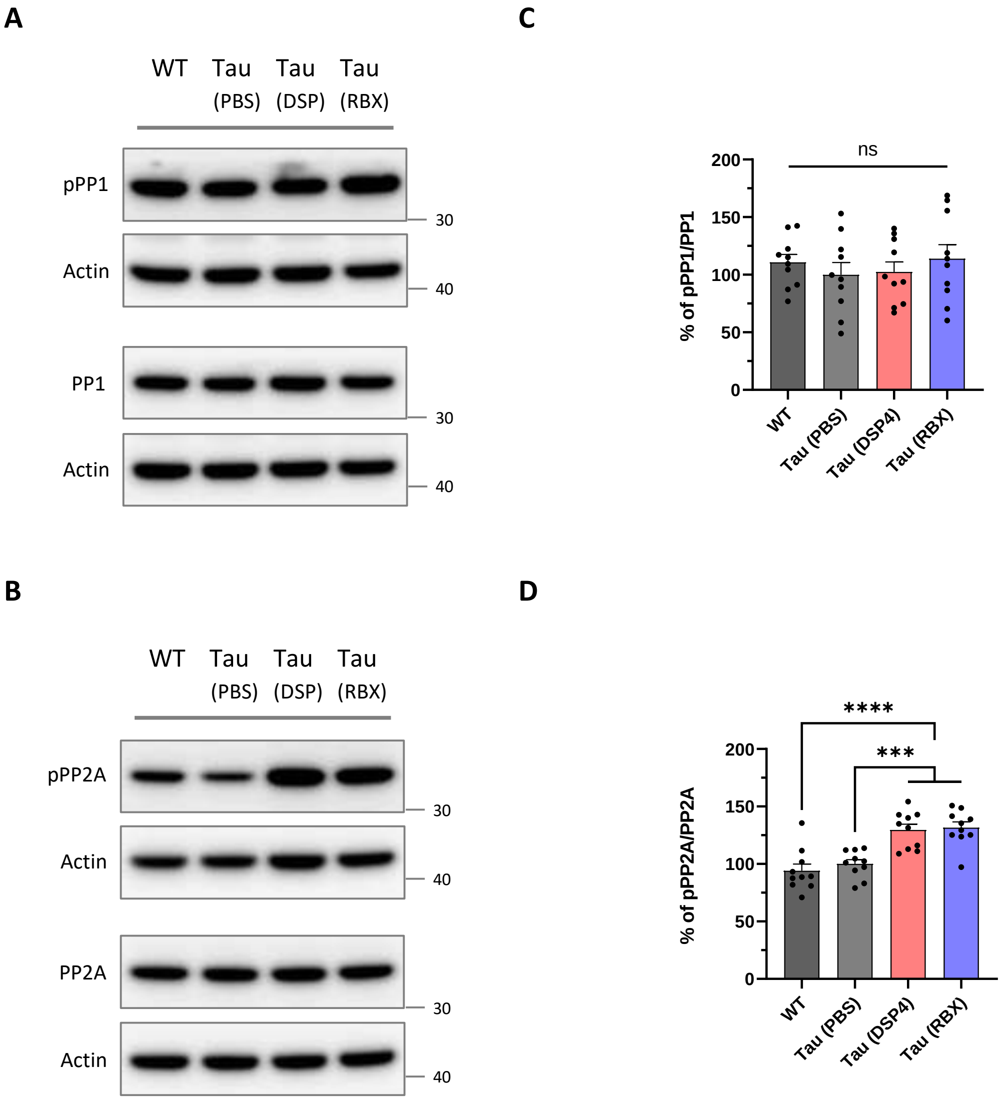

Supplementary Fig. 11

Both DSP4 and RBX reduce PP2A activity. **A-B** Representative western blot images. **C-D** Quantification of specific antibodies against pPP1, PP1, pPP2A, or PP2A in the hippocampus following treatments in the ADLP^{Tau} mice. One dot in the bar graphs represents each mouse (**C-D**). All data represent mean±SEM. Significance was determined by one-way ANOVA and was denoted by *p < 0.05, **p < 0.01, ***p < 0.001, or ****p < 0.0001.

A

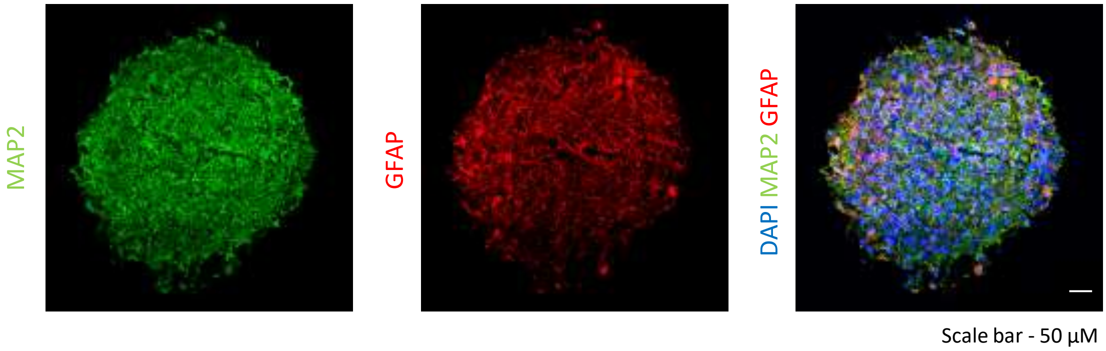

B

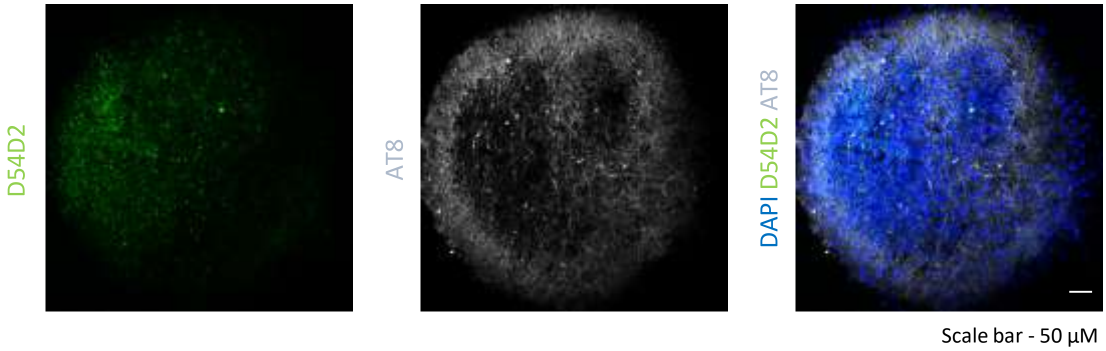

Supplementary Fig. 12

Completed human brain organoids are characterized by IHC. **A-B** Representative immunofluorescence images. Specific antibodies against MAP2, GFAP, D54D2, and AT8 were applied for the images.
